# The hemodynamic effects of intravenous paracetamol (acetaminophen) vs normal saline in cardiac surgery patients: A single center placebo controlled randomized study

**DOI:** 10.1371/journal.pone.0195931

**Published:** 2018-04-16

**Authors:** Elizabeth Chiam, Rinaldo Bellomo, Leonid Churilov, Laurence Weinberg

**Affiliations:** 1 Department of Surgery, The University of Melbourne, Austin Hospital, Heidelberg, Victoria, Australia; 2 Department of Intensive Care, Austin Hospital, Heidelberg, Victoria, Australia; 3 The Florey Institute of Neuroscience and Mental Health, Melbourne Brain Centre, Heidelberg, Victoria, Australia; 4 Anesthesia, Perioperative and Pain Medicine, The University of Melbourne, Victoria, Australia; VU university medical center, NETHERLANDS

## Abstract

The hemodynamic effects of intravenous (IV) paracetamol in patients undergoing cardiac surgery are unknown. We performed a prospective single center placebo controlled randomized study with parallel group design in adult patients undergoing elective cardiac surgery. Participants received paracetamol (1 gram) IV or placebo (an equal volume of 0.9% saline) preoperatively followed by two postoperative doses 6 hours apart. The primary endpoint was the absolute change in systolic (SBP) 30 minutes after the preoperative infusion, analysed using an ANCOVA model. Secondary endpoints included absolute changes in mean arterial pressure (MAP) and diastolic blood pressure (DPB), and other key hemodynamic variables after each infusion. All other endpoints were analysed using random-effect generalized least squares regression modelling with individual patients treated as random effects. Fifty participants were randomly assigned to receive paracetamol (n = 25) or placebo (n = 25). Post preoperative infusion, paracetamol decreased SBP by a mean (SD) of 13 (18) mmHg, p = 0.02, compared to a mean (SD) of 1 (11) mmHg with saline. Paracetamol decreased MAP and DBP by a mean (SD) of 9 (12) mmHg and 8 (9) mmHg (p = 0.01 and 0.02), respectively, compared to a mean (SD) of 1 (8) mmHg and 0 (6) mmHg with placebo. Postoperatively, there were no significant differences in pressure or flow based hemodynamic parameters in both groups. This study provides high quality evidence that the administration of IV paracetamol in patients undergoing cardiac surgery causes a transient decrease in preoperative blood pressure when administered before surgery but no adverse hemodynamic effects when administered in the postoperative setting.

## Introduction

Paracetamol (also known as acetaminophen) is ubiquitously used in hospitals as an antipyretic and analgesic. It is frequently administered intravenously (IV) to patients undergoing major surgery and to critically ill patients, where oral intake and rectal suppositories may not be possible [1]. Intravenous paracetamol is routinely used in our institution preoperatively and postoperatively following cardiac surgery as a component of multimodal postoperative pain therapy and enhanced recovery after surgery. Unlike non-steroidal anti-inflammatory drugs, paracetamol poses no increased risks of surgical site and upper gastrointestinal bleeding. Additionally, prophylactic use of IV paracetamol can reduce postoperative nausea and vomiting [2] and morphine use [3]. For these reasons, paracetamol appears a safe and attractive analgesic adjunct in cardiac surgical patients.

However, in both a healthy volunteer setting [4], in critically ill patients [5–13], clinical data suggests that IV paracetamol may cause hypotension. This may have important clinical implications for patients undergoing cardiac surgery where perioperative hemodynamic instability and hypotension are common. Despite these concerns, there no studies have evaluated the hemodynamic effects of IV paracetamol in the context of cardiac surgery. Therefore, we conducted a prospective, double-blind, single center, placebo controlled randomized study with a two-arm parallel group design to test the hypothesis that IV paracetamol lowers preoperative blood pressure in cardiac surgical patients when compared to placebo.

## Materials and methods

The study design and final Trial protocol was completed on 26^th^ March 2013. The Austin Health Research and Ethics Committee approved this study on June 6^th^ 2013 (number: 05006/2013). The first participant was enrolled on 7^th^ June 2103 and the last participant enrolled on 19^th^ November 2014. We registered the study with the Australian New Zealand Clinical Trials Registry on 19^th^ October 2015 (number: ACTRN12615001099516). Registration was approved retrospectively due to an inadvertent and genuine error in submission. The authors confirm that all ongoing and related trials for this drug/intervention are registered.

The study was conducted according to the principles expressed in the Declaration of Helsinki. All participants provided informed written consent. Between 1^st^ July 2013 and 14^th^ October 2014, we conducted a prospective, double-blind, single center, placebo controlled randomized study with a two-arm parallel group design at a university metropolitan hospital with a dedicated cardiac surgery service. Participants were identified from elective cardiac surgery waiting lists. Inclusion criteria included adult patients undergoing elective primary coronary artery bypass grafting (CABG) or valve surgery. Exclusion criteria included administration of paracetamol or non-steroidal anti-inflammatory drugs within 24 hours of surgery, caffeine consumption less than 10 hours prior to surgery, pregnancy, derangement in any liver function test, severe chronic renal impairment (preoperative creatinine >150 μ L^-1^) and morbid obesity (body mass index >35 kg m^-2^).

All patients were fasted two hours for clear fluids and six hours for a light meal. Preoperative data collection was conducted in a dedicated anesthesia monitoring room, with standardized illumination intensity and noise, and with an ambient air temperature set to 21°C. On the day of surgery, an independent clinical pharmacist prepared three 100 mL IV infusions, which contained paracetamol (1 g paracetamol + 3.91 g mannitol/100 mL) (Actavis Australia, The Rocks, NSW, Australia), or saline 0.9% (100 mL) (placebo) (Baxter Healthcare, Toongabbie, NSW, Australia) as a control. On arrival, participants were placed on a standard operating table and administered midazolam IV 0.025 mg kg^-1^ and fentanyl IV 0.5 ug kg^-1^ for mild pre-operative sedation. Local anesthesia solution was used for the insertion of all invasive lines. Using a standardized technique, all patients had a 15cm 20G arterial line (Leardale, Vygon, UK) inserted into their non-dominant brachial artery for continuous blood pressure measurements, and a continuous cardiac output pulmonary artery catheter (CCombo, Edwards Lifesciences, North Ride, NSW) inserted via the right internal jugular vein for continuous cardiac output, central venous (CVP) and pulmonary artery pressure measurements. After the insertion of invasive lines, participants were placed 15-degree head up, with their head resting on a pillow for comfort. The use of a forced air warming device, IV fluids, or any other medications, were prohibited to avoid confounders.

Participants were given a 15 minutes stabilisation period before baseline hemodynamic measurements were recorded. The paracetamol or saline infusion was then administered over a 15 minute period. After a 30 minute observation period participants were then transferred into the operating room and underwent anesthesia and surgery as per standard anesthesia and surgical care. Postoperatively, all participants were transferred to the intensive care unit (ICU) for routine care. One hour after arrival two further doses paracetamol or placebo were administered over a 15 minute period, each 6 hours apart. The primary endpoint was the absolute change in systolic blood pressure (SBP) 30 minutes (T30) after the infusion of paracetamol or saline. Secondary endpoints included changes in mean and systolic blood pressures, cardiac index, pulmonary artery pressures, systemic vascular resistance index (SVRI) and heart rate (HR). Pulmonary artery pressure was measured directly from the pulmonary artery catheter. Cardiac index was measured continuously via the continuous cardiac output pulmonary artery catheter. Systemic vascular resistance index was calculated from the MAP, CVP and cardiac index using standardized formulae. Normal reference values for the endpoints were: MAP: 70–105 mmHg; SBP: 100–140 mmHg; DBP: 60–90 mmHg; SVRI: 1970–2395 mmHg L^-1^ min^-1^ m^2^; Cardiac index: 2.5–4.0 L min^-1^ m^2^; HR: 60–90 beats min^-1^.

Measurements were recorded at the following time points after the baseline measurement: i) 5 (T5), 8 (T8), 15 (T15) and 30 (T30) minutes after the start of the preoperative infusion and ii) 15 (T15) and 30 (T30) minutes after the start of the postoperative infusions, with hourly measurements for six consecutive hours. Other data collected included baseline patient characteristics, intraoperative use of fluids and vasoactive medications, cardiopulmonary bypass time, aortic cross-clamp time, surgery duration, postoperative use of fluid and vasoactive medications during, ventilator times, and length of ICU and hospital stay. Study participants, surgeons, anesthetists, nurses and all peri-operative staff were blinded to treatment assignments. The randomization sequence was decoded only after data analysis.

Sample size calculations were based on our institution’s pilot data evaluating patients undergoing cardiac surgery. We used inference for means comparing two independent samples with a t test (https://www.stat.ubc.ca). With a mean preoperative systolic blood pressure of 135 mmHg, and a SD of 10mmHg, assuming two-tailed alpha value of 0.05, the total sample of 36 participants (equally distributed between two arms) would yield 80% power to demonstrate the clinically important difference between the paracetamol and saline group of 10 mmHg, assuming common variance between the groups. To allow for violations or breaches in the study protocol, we received ethics approval to recruit 50 participants. An independent statistician generated a computerized sequence of 50 allocation codes, 25 for each group via 5 randomly permuted blocks of 10 participants each. We used a first plan generator (randomization.com) using treatment labels as “Paracetamol” and Saline” with the initial participant identification number being 1. Independent pharmacy staff received the randomization order and prepared all study drugs along with sealed allocation codes in sequentially-numbered opaque envelopes. All study participants and researchers were blinded to these codes until the end of data collection.

Statistical analysis was performed using commercial statistical software STATA/IC v.13 with a p value of 0.05 to indicate statistical significance. The analysis population was defined as intention to treat. Study endpoints (absolute changes from baseline value to value at 30 min) were analysed using an ANCOVA model. For the primary end point, we used the SBP value at 30 min as an outcome, the treatment group as a factor, and the baseline SBP value as a treatment covariate. In addition, longitudinal data modelled over time were analysed using random-effect generalized least squares regression modelling (GLS) due to the repeated measures nature of the data. Both the effect of treatment adjusted for time and treatment-by-time interaction were estimated using respective GLS models. We tested two separate questions: “what is the average difference in the outcome between the groups (adjusting for time)?”—this is the group effect, and “is the rate of increase/decrease in outcome over time similar or different between two groups?”—this is the time-by-treatment interaction. Model adjustments for patient’s age, body mass index (BMI), gender and Euroscore (for pre-operative dose) and for age, BMI, gender, Euroscore, surgery duration, cardiopulmonary bypass, aortic cross-clamp time, fluid intervention and vasoactive medications (for post-operative doses) were performed to estimate the robustness of the results. The study was reported in accordance with the CONSORT Guidelines for reporting randomized trials [14]. No changes to the methods or outcomes measured were undertaken after trial commencement. No interim analysis was conducted. During data collection, there were no missing observations. The study protocol is presented as S1 Protocol.

## Results

**Fig 1:**
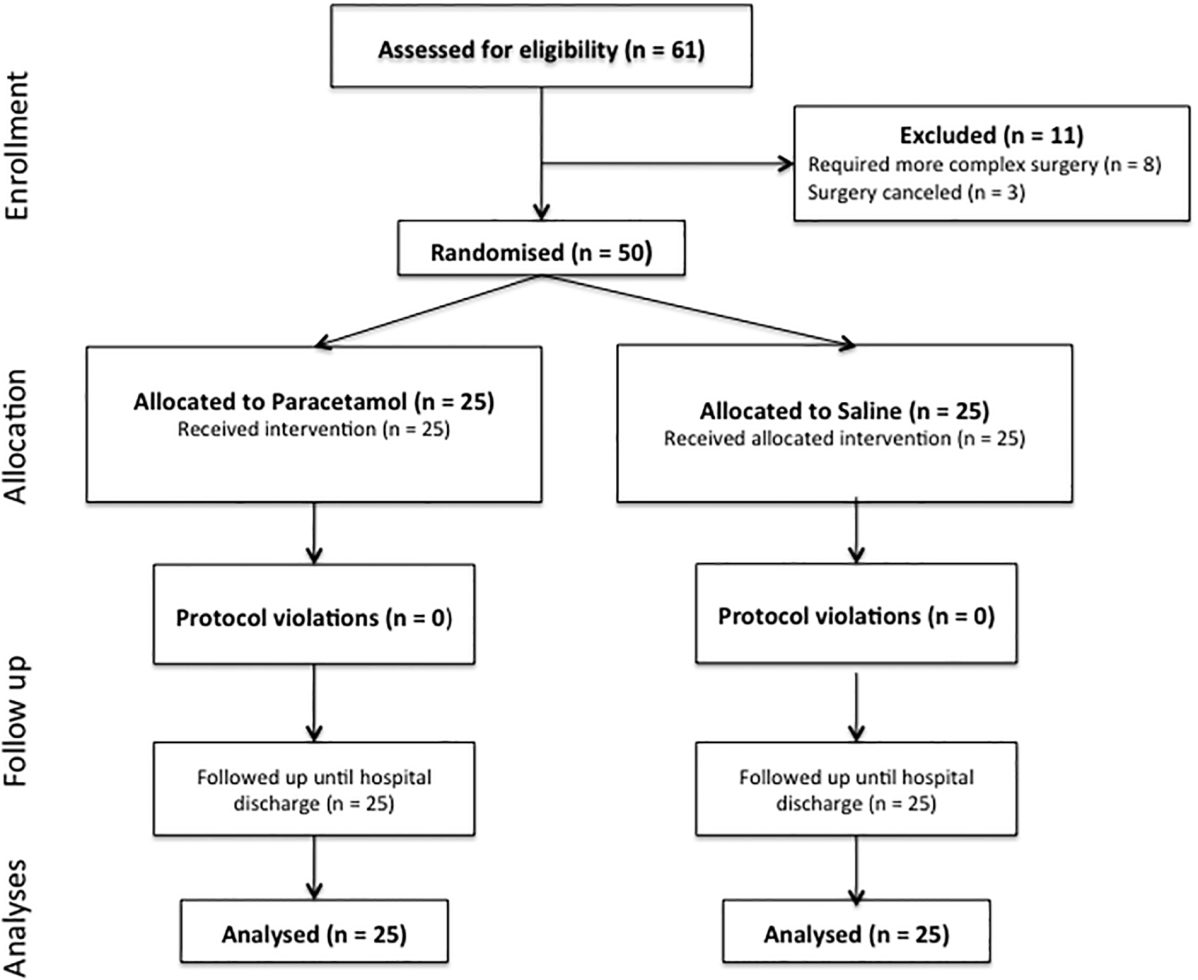
Consort diagram.

Sixty-one participants were screened for eligibility, eight patients required more complex cardiac operations and were withdrawn, and three patients had surgery cancelled. Twenty-five participants were randomized to IV paracetamol and twenty-five participants to saline (Fig 1). There were no violations or breaches of the study protocol. Baseline characteristics, operation type, co-morbidities, and Euroscore were similar in both groups (Table 1). There were no significant differences with regard to surgical duration, cardiopulmonary bypass time, use of fluids, and vasoactive medications (Table 1). The CONSORT checklist is presented as S2 checklist. The raw dataset is presented as S3 dataset.

**Table 1:** Perioperative participant characteristics. Data is mean (standard deviation) or number (proportion).

Preoperative characteristics	Paracetamol (N = 25)	Saline (N = 25)	
Age (years)	66.9 (11.9)	67.8 (10.4)	
Male gender	20 (80%)	22 (88%)	
Weight (kg)	82.3 (16.6)	88.9 (17.8)	
Height (cm)	172.2 (0.2)	172.3 (9.7)	
**Type of surgery**			
Coronary artery bypass graft	16 (64%)	15 (60%)	
Aortic valve replacement	5 (20%)	8 (32%)	
Mitral valve repair	4 (16%)	2 (8%)	
**Coexisting conditions**			
Diabetes mellitus	15 (60%)	16 (64%)	
Peripheral arterial vascular disease	18 (72%)	20 (80%)	
Hypertension	21 (84%)	19 (76%)	
Chronic obstructive pulmonary disease	10 (40%)	9 (36%)	
Mild chronic renal impairment	10 (40%)	11 (44%)	
Euroscore II	1.1 (0.6)	1.5 (1.4)	
**Intraoperative variables**			**P value**
Surgery duration (mins)	252.9 (48.9)	257.1 (54.1)	0.8
Cardiopulmonary bypass time (mins)	92.6 (21.9)	99.4 (35.7)	0.4
Intraoperative fluid (mL)	2015 (879)	2209 (1107)	0.5
Intraoperative noradrenalin (ug)	86 (140.3)	192 (323.2)	0.1
Postoperative fluid (mL)	3548 (1553)	3213 (1177)	0.4
Postoperative noradrenalin (ug)	1289 (2071)	1682 (2470)	0.5

**Fig 2:**
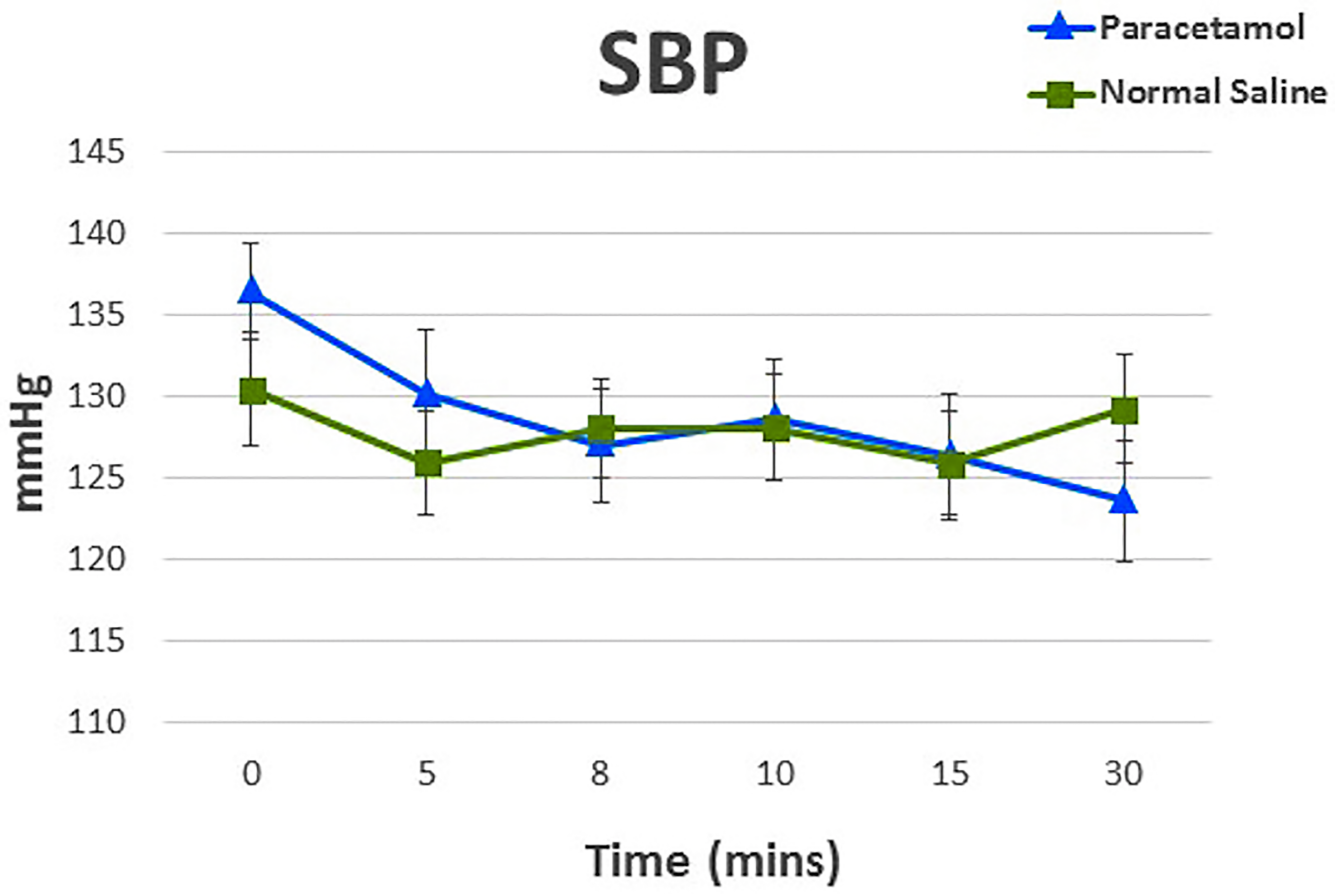
Preoperative changes in systolic blood pressure (SBP) in participants receiving paracetamol or saline before cardiac surgery. Values are mean (SD).

Regarding the primary end point, the absolute mean (SD) change in systolic blood pressure 30 minutes after the infusion of paracetamol or saline was -13 (18) mmHg and for saline was -1 (11) mmHg. The mean between-group difference (estimated from the linear mixed effects model) in absolute change at 30 min adjusted for the baseline values was -11 mmHg (95%CI -20 to -2; p = 0.02). There were no significant changes in SBP between the groups after adjustments for age, BMI, gender and Euroscore. Between baseline and 30 minutes after the infusion, there were a total of 14 hypotensive events i.e. reduction of ≥ 20% SBP from the baseline value, all of which occurred in paracetamol group. These events were self-limiting in all but 2 patients, who both required treatment with a single dose of intravenous metaraminol (0.5mg). Changes in SBP are presented graphically in Fig 2, and summarized in Table 2.

**Table 2:** Primary and secondary endpoints analysed using an ANCOVA model. Data presented as absolute changes from baseline to 30 min.

	Saline		Paracetamol				
	Baseline	Absolute Change	Baseline	Absolute Change	Mean between-group difference in absolute change	95% CI	*p* value (adjusted between-group difference)
SBP* **(mmHg) (**Primary outcome)	130 (24)	-1 (11)	136 (21)	-13 (18)	-11	-20 to -2	0.02
**DBP (mmHg)**	61 (10)	0 (6)	67 (11)	-8 (9)	-6	-11 to -2	0.01
**MAP (mmHg)**	87 (17)	-1 (8)	90 (12)	-9 (12)	-7	-12 to -1	0.02
**sPAP (mmHg)**	31 (11)	1 (7)	27 (9)	2 (7)	1	-3 to 5	0.65
**dPAP (mmHg)**	14 (7)	1 (5)	15 (6)	1 (5)	0	-3 to 3	0.99
**mPAP (mmHg)**	23 (13)	-1 (5)	19 (8)	1 (5)	-0.3	-3 to 3	0.83
**CVP (mmHg)**	7 (5)	1 (3)	9 (5)	-1 (5)	-1.5	-4 to 1	0.16
**HR (beats min**^**-1**^**)**	66 (12)	-2 (7)	72 (13)	-1 (6)	2	-1 to 6	0.21
**CI (L min**^**-1**^ **m**^**2**^**)**	2.49 (1.00)	0 (1)	2.72 (1.02)	0 (1)	0.1	-0.2 to 0.5	0.41
**SVRI (dynes sec**^**-1**^ **cm**^**-1**^ **m**^**2**^**)**	2746 (680)	-166 (493)	2695 (1027)	-332 (617)	-182	-465 to 102	0.20

*ANCOVA model with the SBP value at 30 min as an outcome, the treatment group as a factor, and the baseline SBP value as a treatment covariate.

**Fig 3:**
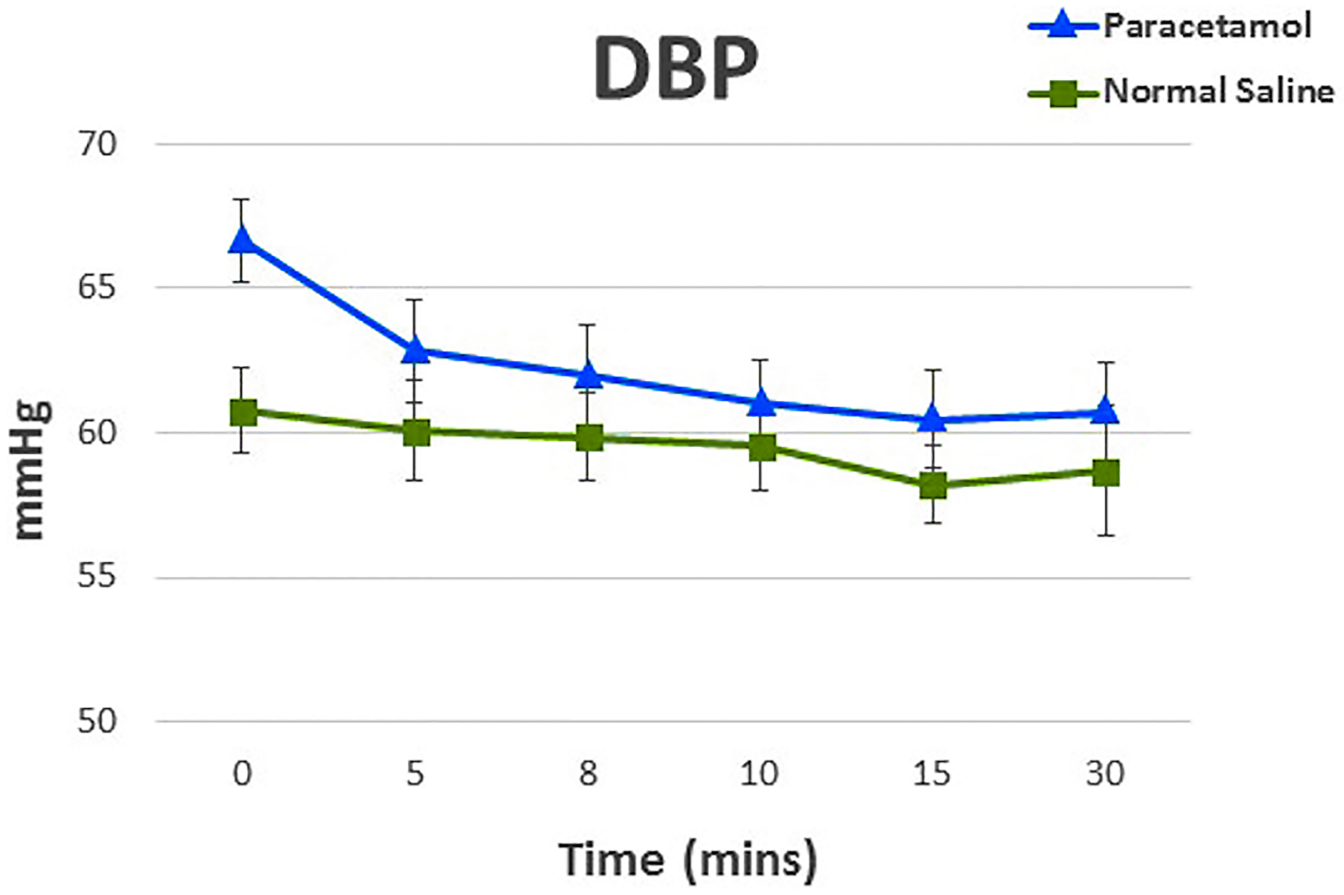
Preoperative changes in diastolic blood pressure (DBP) in participants receiving paracetamol or saline before cardiac surgery. Values are mean (SD).

**Fig 4:**
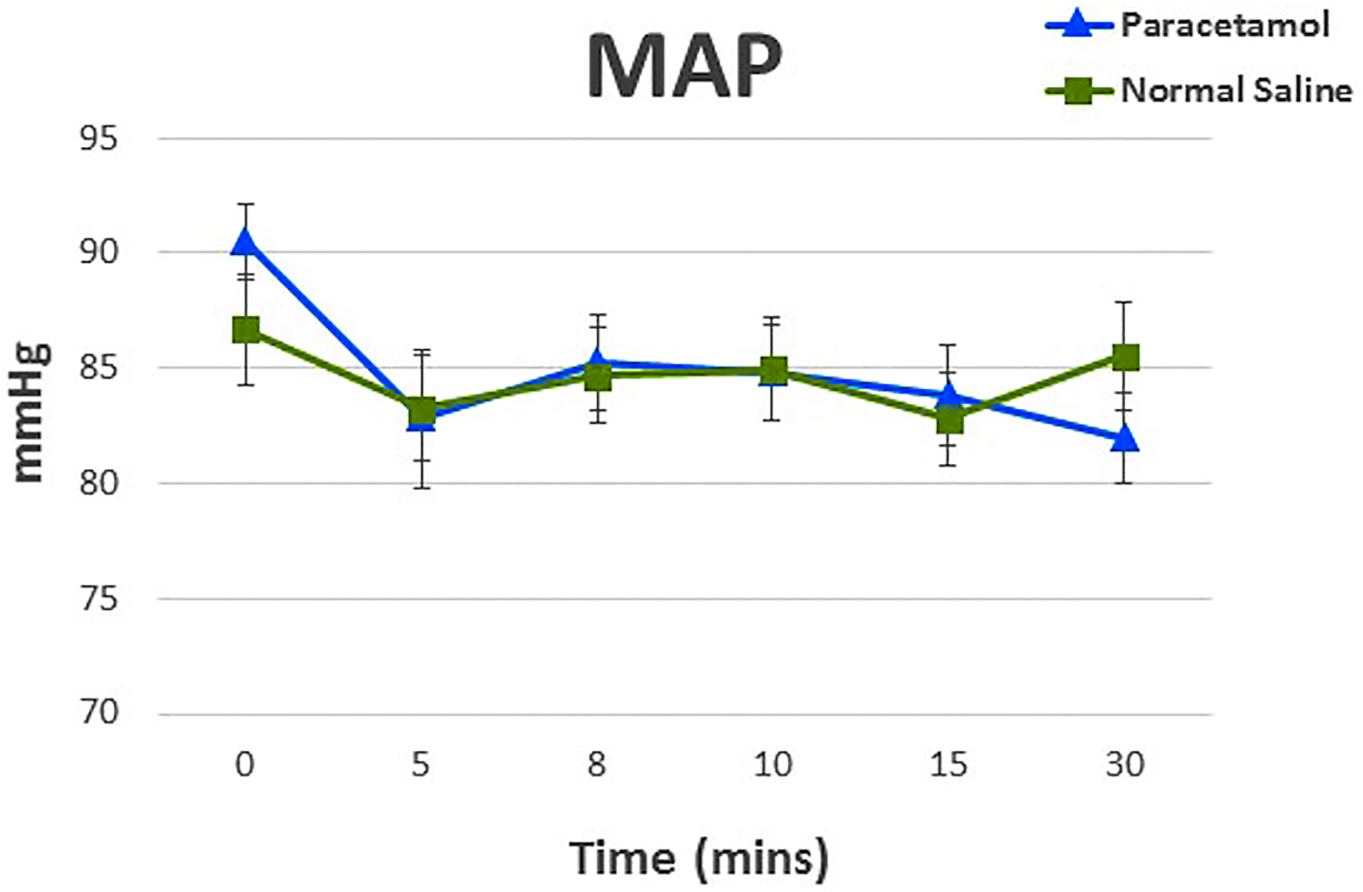
Preoperative changes in mean arterial pressure (MAP) in participants receiving paracetamol or saline before cardiac surgery. Values are mean (SD).

Paracetamol also decreased MAP and DBP by a mean (SD) of 9 (12) mmHg and 8 (9) mmHg compared to a mean (SD) of 1 (8) mmHg and 0 (6) mmHg, respectively with saline (p = 0.01 and 0.02). SVRI decreased by 332 dynes sec^-1^ cm^-5^ m^2^ in the paracetamol group compared to 166 dynes sec^-1^ cm^-5^ m^2^ in the saline group (p = 0.20). Analyses of cardiac index, pulmonary artery pressures, SVRI, and HR showed no statistical significance in their interaction between the treatments over time. Changes DBP and MAP after the preoperative dose from baseline to the 30 minute (T30) endpoint are presented graphically in Figs 3 and 4, and in summarized in Table 2. There were no significant changes in mean arterial and diastolic blood pressure between the groups after adjustments for age, BMI, gender and Euroscore. Longitudinal data of endpoints during and after the 15-minute infusion of paracetamol preoperatively are presented in S4 Table.

After the two postoperative infusions of paracetamol there were no significant differences in pressure or flow based hemodynamic parameters compared to saline. Analyses of cardiac index, pulmonary artery pressures, SVRI, and HR also showed no statistical significance in their interaction between the treatments over time. After adjustments for age, BMI, gender, Euroscore, surgery duration, cardiopulmonary bypass time, aortic cross-clamp time, use of fluids and vasoactive medications, there were still no hemodynamic differences between the groups. Longitudinal data of the endpoints of the infusion of paracetamol after cardiac surgery are presented in S5 and S6 Tables. The mean (SD) length of ICU stay was 47.0 (22.4) hours in the paracetamol group and 43.8 (26.7) hours in the saline group (p = 0.13). Duration of hospital stay was 8.8 (4.1) days in the paracetamol group vs. 10.4 (6.9) days in the saline group (p = 0.03).

## Discussion

We performed a randomized blinded controlled trial comparing the effects of IV paracetamol and placebo (saline) on hemodynamics during and after cardiac surgery. This study provides plausible physiological evidence that the preoperative administration of 1 gram IV paracetamol administered before induction of anesthesia in patients awaiting cardiac surgery, causes a transient decrease in preoperative blood pressure 30 minutes after the start of infusion. However, administration of two consecutive six hourly paracetamol doses given after surgery, caused no significant hemodynamic adverse effects, findings similar to Avellaneda et al., who administered 1 gram of propacetamol (a prodrug of paracetamol) after tracheal extubation post-elective cardiac surgery and observed no clinically significant impairment in hemodynamic function [15].

Recently, the use of IV paracetamol in the context of cardiac surgery has been examined. Douzjian and Kulik [16] provided a systemic review of the literature including results from the pro-drug, propacetamol. However, the authors were unable to form a meta-analysis of the studies given the lack of similarities between the nine cardiac surgery trials they investigated. Despite this and the conflicting results of the studies, the authors concluded that IV paracetamol provided minimal clinical benefit to patients and encouraged clinicians to consider limiting their use of IV paracetamol in cardiac surgical patients. Mamoun et al. [17] tested the efficacy of the analgesic effect of IV paracetamol vs. normal saline in cardiac surgery patients. They concluded that IV paracetamol reduced postoperative pain, however failed to reduce postoperative opioid use. Hemodynamic effects of paracetamol were not examined.

In the context of critical illness, multiple clinical studies have suggested that IV paracetamol may cause hypotension [5–13]. Most of these studies, however, have been limited by their retrospective design, small patient numbers, and lack of randomization and blinding. In addition, as many of these studies were conducted in critically ill patient cohorts, use of vasopressors may have masked the magnitude of the hypotension [6, 7, 9, 10]. More recent investigations of the hemodynamic effects of IV paracetamol have yielded results that suggest that paracetamol is unlikely to induce clinically significant hemodynamic changes [4, 8]. Needleman et al. studied the effects of a rapid infusion of IV paracetamol in ambulatory surgical patients [8] and monitored blood pressure during and after a rapid infusion. Whilst, the administration of paracetamol caused a decrease in blood pressure, these alterations were considered not to be clinically significant. A major limitation of this study, however, was a short observation period of 5 minutes, which may not accurately represent the full period of time when hypotension may occur. In addition, concurrent use of fluid and vasoactive drugs may also confound the intra- and postoperative hemodynamic effects of paracetamol. In our study, the absence of hemodynamic effects observed during and after surgery may be due to extensive perioperative use of vasopressor therapy, which all study participants received as part of standard care.

In contrast, in the context of critical illness, Krajcova et al., found IV paracetamol produced a reduction of MAP. However, there were only 6 patients in this study [12]. More recently our group investigated the hemodynamic effects of IV paracetamol in a healthy volunteer setting [4]. In a blinded, randomized, crossover study, we infused paracetamol over a 15 minute infusion time followed by a 45 minute observation period. Similar to the results of Needleman et al. [8], paracetamol caused a significant decrease in blood pressure immediately after infusion. The possible trigger for the decreased blood pressure was vasodilatation with a decrease in SVRI with no significant changes to cardiac index. These hemodynamic effects were transient, of limited magnitude, and of questionable clinical significance. We postulated that these effects might have a different intensity and duration in patients who are prone to hypotension, such as patients undergoing cardiac surgery.

We have demonstrated in the present study, that similar to our previous findings [4] and those of Krajcova et al. [12], IV paracetamol causes a transient decrease in blood pressure with an accompanying decrease in SVRI during the preoperative infusion period. These findings are of particular interest given that we were able to replicate our findings in a cardiac surgical setting, with greater magnitude of change, possibly a reflection of using more accurate and continuous invasive hemodynamic measurements from the arterial line, central venous catheter, and pulmonary artery catheter, compared to the non-invasive hemodynamic technology used in our previous healthy volunteer study [4]. Importantly we have also demonstrated that, in contrast to the findings of Krajcova et al. [12], the administration of paracetamol failed to have any significant effect on CI as measured by the PAC.

Finally, in a recent large randomized controlled trial regular paracetamol was given to critically ill patients with fever or suspected infection [18] however, detailed hemodynamic data were not collected but shorter length of stay was detected among survivors, a finding similar to our study, where treatment with IV paracetamol was associated with a shorter duration of hospital stay. This shorter length of ICU stay may be due to the synergistic effects paracetamol has with opioid drugs in treatment of postoperative pain management [3, 19, 20] and its reported shorter average time to extubation [20]. Our findings may be relevant beyond cardiac surgery itself. Appreciation of the hemodynamic effects of paracetamol is now important to clinicians in general, as use of IV paracetamol is ubiquitous in the critical care, perioperative and general medical settings, where it is widely used as both an analgesic and antipyretic [21]. In this regard, knowledge of the likely effects of IV paracetamol on blood pressure should permit better choice of timing of infusion and correct diagnostic assessment of the etiology of hypotension in the setting of IV administration.

Our study has several methodological strengths compared to previous studies. It is blinded and randomized, thus minimizing allocation, selection and assessment bias and increasing internal validity. All hemodynamic variables measured were assessed invasively and were not amenable to ascertainment bias or derivation. The concealment of the intervention to all clinicians further decreased bias. The standardisation of study setting during the pre-operative period was strictly adhered to in all patients, ensuring accuracy of hemodynamic data in the absence of analgesic, anesthetic, vasoactive drugs and IV fluids, all of which could confound the measurements of blood pressure. Finally, we used robust statistical methods to adjust for important variables that frequently impact on blood pressure in a cardiac surgical setting. There are also come limitations to our study. First, observations were only continued for 30 minutes after the first administration preoperatively. Extending this study time beyond 30 minutes would have delayed surgery, therefore was not approved by our ethics committee. As evident from Fig 2, SBP drop hasn’t quite reached a nadir at 30 minutes; is it possible that the overall effect maybe greater than described. We do not know whether our results are applicable to more complex cardiac surgery, other scheduled (or emergency) types of operations, or to older, sicker or morbidly obese patients. However, it seems likely that if effects are seen with elective coronary artery surgery similar or even greater effects might be seen with other types of surgery. We also cannot extrapolate our paracetamol infusion results to different combinations of rate, duration or route. However, the dose and speed of administration in our patients reflect practice and recommendations. The findings with IV paracetamol are likely not relevant to oral or per-rectum paracetamol, where the absorption is slower and any hemodynamic effects that might exist are attenuated. We cannot provide information on the mechanism of paracetamol-associated hypotension. However, we have previously demonstrated in volunteers that the hypotensive effect does not appear related to the mannitol present in the IV solution as excipient. Finally, we only detected an effect in the pre-operative setting but not in the post-operative setting. This suggests a greater effect of IV paracetamol in the setting of recent pre-medication or an attenuation of the response to IV paracetamol following a previous dose, or both effects and additional characteristics of ICU treatment or post-operative care, which might attenuate the putative vasodilatory effect of the drug.

## Conclusion

In summary, in a randomized blinded trial comparing the effects of IV paracetamol and placebo (saline) on hemodynamics before and after cardiac surgery, we found that the preoperatively administration of paracetamol caused a transient decrease in blood pressure, but that six-hourly postoperative administrations caused no significant hemodynamic adverse effects. Our findings provide plausible physiological evidence that the administration of IV paracetamol should be used cautiously before cardiac surgery but can be safely used when clinically indicated after elective cardiac surgery, without adversely impacting hemodynamics.

## Supporting information

S1 Protocol Study protocol. (PDF)

S1 Checklist CONSORT checklist. (DOC)

S1 Dataset Dataset. (XLSX)

S1 Table Longitudinal data of endpoints during and after a 15-minute infusion of intravenous paracetamol administered preoperatively. Data analysed using a random-effect generalized least squares regression model.
(DOCX)

S2 Table Longitudinal data of endpoints after intravenous paracetamol administered immediately after cardiac surgery. Data analysed using a random-effect generalized least squares regression model. Changes in postoperative.
(DOCX)

S3 Table Longitudinal data of endpoints after intravenous paracetamol administered 6 hours after cardiac surgery. Data analysed using random-effect generalized least squares regression model.
(DOCX)
