# Supplementary material for: The hemodynamic effects of intravenous paracetamol (acetaminophen) vs normal saline in cardiac surgery patients: A single center placebo controlled randomized study
Source: PLoS One. 2018 Apr 16;13(4):e0195931. doi: 10.1371/journal.pone.0195931 (PMC5901786; doi:10.1371/journal.pone.0195931)
Supplement: S1 Protocol — (PDF) [file pone.0195931.s001.pdf]

PROTOCOL

Haemodynamic Effect of Intravenous Paracetamol during and after Cardiac Surgery

Protocol Number: 1
Version: 1
Date: 26/03/2013

Authors:
Elizabeth Chiam
Laurence Weinberg
Rinaldo Bellomo

Sponsor/s: Nil

Statement of Compliance

This document is a protocol for a research project. This study will be conducted in compliance with all stipulation of this protocol, the conditions of the ethics committee approval, the NHMRC National Statement on ethical Conduct in Human Research (2007) and the Note for Guidance on Good Clinical Practice (CPMP/ICH-135/95).

TABLE OF CONTENTS

CONTENTS

Table of Contents 2

1. Glossary of Abbreviations & Terms 4

2. Study Sites 5

2.1 Study Location/s 5

3. Introduction/Background Information 6

3.1 Lay Summary 6

3.2 Introduction 7

3.3 Background information 8

4. Study Objectives 15

4.1 Hypothesis 15

4.2 Study Aims 16

4.3 Outcome Measures 17

5. Study Design 18

5.1 Study Type & Design & Schedule 18

5.2 Standard Care and Additional to Standard Care Procedures 20

5.3 Randomisation 21

5.4 Study methodology 23

6. Study Population 24

6.1 Recruitment Procedure 24

6.2 Inclusion Criteria 24

6.3 Exclusion Criteria 24

6.4 Consent 24

7. Participant Safety and Withdrawal 25

7.1 Risk Management and Safety 25

7.2 Handling of Withdrawals 25

8. Statistical Methods 25

8.1 Sample Size Estimation & Justification 25

8.2 Power Calculations 25

Results from OpenEpi, Version 2, open source calculator--SSMean 26

8.3 Statistical Methods To Be Undertaken 26

9. Storage of Blood and Tissue Samples 27

9.1 Details of where samples will be stored, and the type of consent for future use of samples 27

10. Data Security & Handling 27

10.1 Details of where records will be kept & How long will they be stored 27

10.2 Confidentiality and Security 27

11. Appendix 29

12. References 30

Glossary of Abbreviations & Terms

Abbreviation	Description (using lay language)
CI	Cardiac Index
CO	Cardiac Output
COX	Cyclooxygenase. Subgroups of COX include COX-1, COX-2 and COX-3
CPB	Cardiopulmonary Bypass
EUROscore	European System for Cardiac Operative Risk Evaluation
FDA	Food and Drug Administration (USA)
MAP	Mean Arterial Pressure
NSAID	Non-Steroidal Anti-Inflammatory Drug
NOS	Nitric Oxide Synthase
Parsonnet indices	The scoring system for predicting risk in cardiac surgery
PG	Prostaglandin
SBP	Systolic Blood Pressure
TGA	Therapeutic Goods Administration (Australia)
TXA	Thromboxane
5HT	Serotonin is a hormone and neurotransmitter that acts. Activation of the 5-HT neurons in the brain stem, releases descending projections to the spinal cord and exert an inhibitory (analgesic) effect on the incoming pain signal. Common serotonin receptors include: $5HT_{1B}$, $5HT_{2A}$, $5HT_{2C}$

1. STUDY SITES

1.1 STUDY LOCATION/S

Site	Address	Contact Person	Phone	Email
Austin Hospital	Studley Road, Heidelberg, Victioria, 3084	Prof Rinaldo Bellomo	03 94965992	Rinaldo.Bellomo@austin.org.au
Austin Hospital	Studley Road, Heidelberg, Victioria, 3084	Dr Laurence Weinberg	03 94965000	Laurence.Weinberg@austin.org.au
Austin Hospital	Studley Road, Heidelberg, Victioria, 3084	Elizabeth Chiam	03 94965468	E.Chiam@student.unimelb.edu.au

2. INTRODUCTION/BACKGROUND INFORMATION

2.1 LAY SUMMARY

Background: Intravenous paracetamol is ubiquitously used in hospitals as an antipyretic and analgesic worldwide. It is administered to a wide array of patients including those undergoing major surgeries, such as cardiac surgery due to its minimal side effect profile. However, recent studies have provided evidence that paracetamol may cause hypotension in critically ill patients, which may have important clinical implications for patients undergoing surgery. The mechanism for this hypotension is unknown but may be attributable to one of the stabilizing compounds found in the formulation, mannitol. Mannitol is a known diuretic and even in small quantities can cause episodes *of* transient hypotension [1]. The paucity of clinical data in this setting justifies the need for further investigation into the safety and efficacy of paracetamol during and after cardiac surgery.

There are currently no double-blinded, randomised controlled trials that assess the haemodynamic effects of paracetamol in patients undergoing cardiac surgery. The effects of cardiopulmonary bypass (CPB) on paracetamol pharmacokinetic are also poorly understood. This study will add to a growing body of evidence evaluating the safety and efficacy of paracetamol use in patients undergoing cardiac surgery.

Hypothesis: Paracetamol (1g IV) has adverse effects on blood pressure in patients undergoing cardiac surgery.

Primary endpoint: Changes in systolic, diastolic and mean arterial pressure 15 minutes after infusion of paracetamol.

Secondary endpoints: Changes in cardiac output and cardiac index, core body temperature, time to first post-operative request for opioid treatment, amount of opioid administered during stay in ICU, frequency of inotrope use, renal biomarkers (serum and urine NGAL and cystatin C) and pro-inflammatory effects (TNFα, IL-6 and IL-10).

Other variables measured: Plasma paracetamol concentrations immediately prior to infusion, 15 minutes, 30 minutes post infusion, immediately pre-cardiopulmonary bypass (CPB) and post-CPB.

Other data collected: Patient demographics, type of surgery, CPB time, total volume of administered CPB prime fluids, EuroSCORE, Parsonnet indices, duration of ICU stay, duration of hospital stay and adverse events.

No of patients: 50

No of recruiting hospitals: 1

Randomisation: Patients will be randomised in a 1:1 fashion via a computer generated randomisation program to either paracetamol IV formulation (N=25) or control treatment (normal saline 0.9%) (N=25).

Blinding: This is a double-blinded clinical trial. Surgical teams, intraoperative and postoperative nursing staff, and patients will be blinded to assignment of treatment.

2.2 INTRODUCTION

Paracetamol is one of the most commonly used medications in the world. This can be attributed to its ubiquitous use in both prescription and non-prescription drugs. Annually, over 200 million paracetamol-containing prescriptions are filled in the USA and non- prescription sales exceed 25 billion doses per year, making it the most commonly dispensed pharmaceutical in America [2, 3]. At the recommended doses, paracetamol seems to possess effective antipyretic and analgesic effects and with limited adverse events it is considered safe for public use. While paracetamol is a leading over-the-counter medicine, its use in hospitals is also well-established.

Surprisingly, given the worldwide ubiquitous use of paracetamol, prospective controlled studies are limited and the results are very inconsistent [4-7]. Importantly, emerging clinical data has suggested that intravenous (IV) paracetamol has a propensity to cause hypotension in critically ill patients [8, 9]. This may have important clinical implications for patients undergoing cardiac surgery. The paucity of clinical data in this setting justifies the need for further investigation into the safety and efficacy of IV paracetamol during and after cardiac surgery.

We plan to formally examine the haemodynamic and pharmacokinetic effects of paracetamol in cardiac surgery patients. We will conduct a double-blinded randomised controlled study to ascertain whether:

- Paracetamol has any haemodynamic effects during and after cardiac surgery and evaluate the effects of cardiopulmonary bypass on paracetamol pharmacokinetics.

In addition, we will evaluate if paracetamol has:

- Any antipyretic effects during and after cardiac surgery
- Any analgesic properties and opioid sparing effect

2.3 BACKGROUND INFORMATION

The following table lists the recommended dosage and general pharmacokinetic properties of IV paracetamol.

Table 1: Prescribing summary of IV paracetamol [10]

**Indication:**	
Short-term treatment of moderate pain and fever when administration by the intravenous route is clinically justified	
**Paracetamol structural formula**	
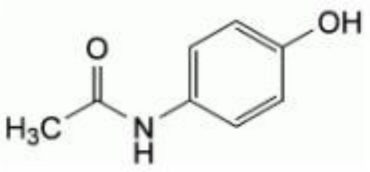<br>Molecular weight: 151.2	Paracetamol is an odourless, white, crystalline solid powder. It is soluble in a variety of common solvents including water, alcohol and acetone. It is available in 3 different formulations (oral, rectal and intravenous). Actavis IV paracetamol is formulated in a clear solution that contains 1g/100mL of paracetamol. The solution also contains mannitol, cysteine hydrochloride monohydrate, dibasic dihydrate sodium phosphate, sodium hydroxide, hydrochloric acid and water for injections.
**Mechanism of action:**	
Thought to act on multiple pain pathways including COX-1, COX-2, COX-3, serotonergic, nitric oxide synthase and cannabinoid pathways	
**Dosage and administration**	
**Adults**	Weight: > 50kg: 1g (max 4g/day), <50kg: 15mg/kg (max 3g/day)
**Duration of infusion**	15 minutes
**Minimum dose interval**	4 hours (8 hours in patients with severe renal failure)
**Availability and storage**	
Glass vials containing 50 or 100 mL of paracetamol 10 mg/mL solution (i.e. 0.5 or 1 g per vial); store at room temperature (≤30°C)	
**Pharmacokinetic properties**	
**Maximum plasma concentration**	Single 1g dose: 30µg/mL
**Volume of distribution**	1L/kg
**Metabolism**	*Therapeutic doses:* predominantly in the liver by glucuronidation and sulfation<br>*Higher doses:* via the cytochrome P450 2E1 pathway, producing *N*-acetyl-*p*-benzoquinone (NAPQI), which rapidly forms conjugates with glutathione to generate nontoxic metabolites<br>*Acute overdose:* glutathione stores may be depleted, NAPQI may accumulate and hepatotoxicity may occur (administer *N*-acetylcysteine as antidote within 10 h of overdose)
**Systemic clearance**	18L/hour
**Mean elimination half-life (adults)**	2.7 hours
**Excretion**	Predominantly in the urine as glucuronide (60–80%) and sulfide (20–30%) conjugates<br><5% as unchanged drug in the urine

PHARMACOLOGICAL EFFECTS OF PARACETAMOL

1. Different routes of paracetamol administration

Paracetamol is available in a variety of formulations each with differing pharmacokinetic profiles. Commonly available oral (PO) and rectal (PR) formulations provide adequate pain relief and treatment of fever, yet their pharmacological value in cardiac surgical patients has not been formally evaluated and its clinical efficacy within this patient demographic has yet to be determined. This is in part due to PO administration being contraindicated in sedated patients in addition to variable absorption rates with the use of PO and PR formulations, thus delayed latency in the time to produce the desired therapeutic effects [11-13]. Some reports have claimed PO and PR were unable to produce the required minimum plasma level of 10mg/mL for antipyretic and analgesic effects [11, 14] and subsequent requirements for supplementary analgesia are common in the post-operative period. A major advancement in the clinical use of paracetamol has been the recent introduction of intravenous formulations. Singla et al. demonstrated the differences in adsorption between PO, PR and IV administration (Figure 1) [14] and identified the potential benefits of IV paracetamol over PO or PR use.

Figure 1. Mean paracetamol (acetaminophen) plasma concentration over time in 6 subjects after intravenous (IV), oral and rectal administration of 1000 mg [14].

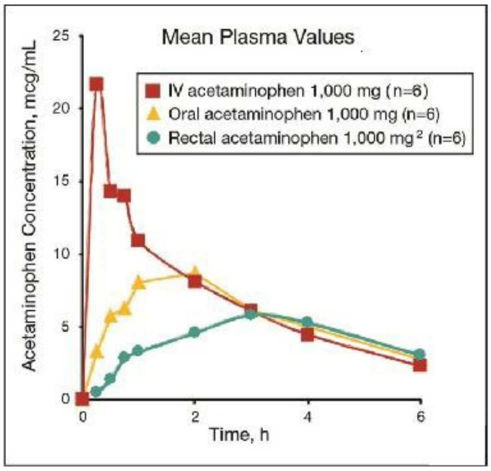

The formulation for IV paracetamol has undergone important changes since its conception in the early 1990s. Propacetamol (a pro-drug of paracetamol) was previously used for intravenous administration of paracetamol. However, it has since been discontinued due to complaints of pain on infusion and the development of contact dermatitis during handling in healthcare professionals [15, 16]. IV paracetamol was launched in the early 2000s in the UK with US FDA and Australian TGA approval in 2010 and 2011 respectively. McNicol et al. provided evidence that IV propacetamol and IV paracetamol were both capable of rapidly

producing therapeutic plasma-drug levels with peak plasma concentrations observed shortly after the 15 minute infusion time [17]. Pain scores were similar between the two groups. They concluded that IV paracetamol mirrors the efficacy of its predecessor, yet avoids the observed side-effects of propacetamol. However this evidence is largely based on observational studies rather than large randomised controlled trials which identify causality. Additionally, there is a marked difference in their formulations which has gone mostly unnoticed. Propacetamol is available in a powdered form necessitating reconstitution

(generally in sodium citrate). In solution, propacetamol is unstable and requires immediate infusion after reconstitution. The currently used preparation of IV paracetamol is ready-to- use and available in solution due to the addition of stabilizing compounds such as mannitol. Mannitol is a known diuretic and even in small quantities can cause episodes of transient hypotension [1]. Further investigation is required to identify whether the 3.91g of mannitol in 100mL IV paracetamol confers haemodynamic changes in high risk patients.

2. Non-opioid analgesia and proposed mechanisms of action of paracetamol

Paracetamol and non-steroidal anti-inflammatory drugs (NSAIDs) are classified as non- opioid drugs. Opioid-related adverse events such as nausea, vomiting, sedation and respiratory depression are seldom observed in paracetamol use. The pharmacodynamics of NSAIDs has been well documented [18-20]. NSAIDs act by inhibiting the cyclooxygenase prostaglandin pathways. Central inhibition of prostaglandin PGE2 provides the observed antipyretic and analgesic effects. It is thought that the anti-inflammatory properties of NSAIDs are largely due to peripheral actions at the site of inflammation. However, unwanted side-effects of NSAIDs occur during COX-1 inhibition of the prostaglandins (PGI_2, PGE_2 and TXA_2) that usually promote healthy gastric mucosal secretions and platelet aggregation. As a result, NSAIDs are known to cause gastrointestinal toxicity (e.g. gastric ulcers) and an increased bleeding time (Figure 2) [21].

Figure 2. Effects of NSAIDs on the cyclooxygenase pathway.

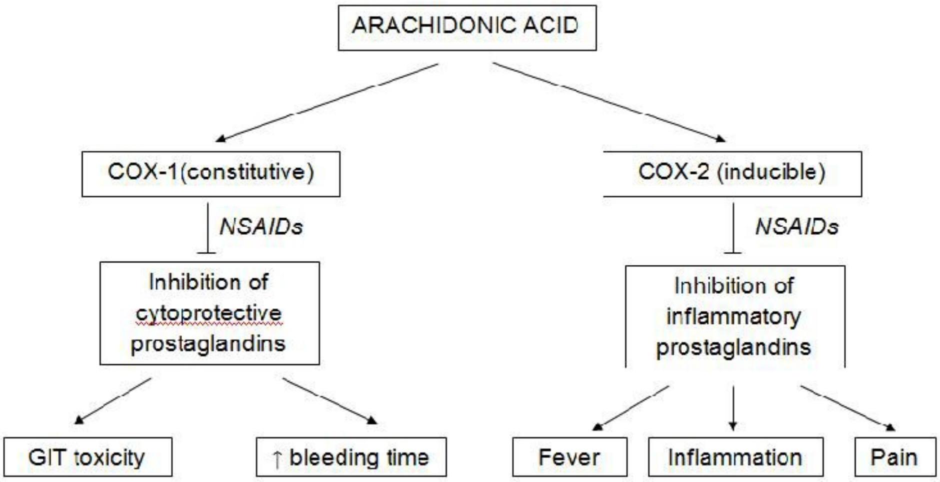

Despite its discovery more than 100 years ago, the exact mechanism of action of paracetamol is still a matter of debate. All non-opioid analgesics have an antipyretic effect but paracetamol lacks both the peripheral anti-inflammatory and anti-platelet response seen with the use of NSAIDs [22]. As paracetamol is similar to Aspirin (a commonly used NSAID) in treating pain and fever, the majority of studies have rationalized that paracetamol acts via the inhibition of the cyclooxygenase pathway. COX-1 has been implicated as the potential cause for analgesic effect of paracetamol in the mouse model. Ayoub et al. found a reduction in antinociceptive activity (the equivalent of an analgesic effect in animal models) in COX-1 knock-out mice [23]. However, hypothermia or antipyretic effects were lacking suggesting paracetamol may not act solely at COX-1. Effects on the COX-2 pathway may

explain the lack of antipyresis missing from the COX-1 theory. A human ex-vivo whole blood study found that paracetamol had comparable antipyretic effects to NSAIDs and selective COX-2 inhibitors [24]. An inconsistency is that paracetamol is not associated with the adverse events profile of common COX-2 inhibitors. Additionally, Flower and Vane [25] discovered that paracetamol had a greater inhibitory effect on COX activity in brain homogenates compared to the spleen which supports the idea that COX variant enzymes exist. With gaps in the COX-1 and COX-2 hypotheses, studies have suggested a possible role of the enzyme variant COX-3 [26]. Little is known about COX-3, which was first discovered in canines. This theory is unlikely to translate well in humans due to mutational differences between canine COX-3 and mouse/human COX-3 sequences [27].

More recent findings have suggested other possible mechanisms including indirect and direct stimulation of the cannabinoid, nitric oxide synthase and serotonergic pathways [28]. Table 3 summarizes the postulated mechanisms of action. However, the overall consensus is that paracetamol has a central site of action with little if any peripheral effect. It is likely that paracetamol has a multifactorial mechanism of action which may include the activation of different pain pathways hence the difficulty in elucidating its precise mechanism of action.

Table 3. Summary of the theories suggested explaining the mechanism of action with paracetamol use.

Pain Pathway	Proposed theory	Conflicting evidence
**COX-1**	Antinociceptive effect in mouse model [23]	No antipyretic effect found [23]
**COX-2**	Antipyretic effect in human whole blood samples[29]	Dissimilar adverse events profile compared to other COX-2 inhibitors [30]
**COX-3**	***Rat study:*** paracetamol inhibits COX in brain homogenates supporting theory of the existence of COX enzyme variants e.g. COX-3 [30]	Low expression and kinetic activity in rats [27]<br><br>Mutational difference between canine and human/mouse COX-3. Canine data cannot be translated to human use [27]
**Cannabinoid (Direct and Indirect)**	***Mouse study:*** AM404 (paracetamol prodrug) is a direct and potent agonist of the vanilloid TRPV1. AM404 indirectly acts on the cannabinoid CB1 receptor conferring analgesia and perhaps thermoregulation in the CNS [31]<br><br>***Human study:*** paracetamol shares similar behavioural effects e.g. euphoria, relaxation and tranquillity associated with use of cannabinoids [32, 33]	Requires further evidentiary support in human studies
**Serotonergic/ 5-HT (Indirect)**	***Rat study:*** increase in 5-HT levels in the brain caused downregulation of the 5-HT receptors after administration of paracetamol [34]<br><br>***Rat study:*** paracetamol's ability to produce analgesia is blocked by $5HT_{1B}$, $5HT_{2A}$ and $5HT_{2C}$ receptors antagonists [35]<br><br>***Human study:*** 1000mg oral paracetamol significantly reduced pain caused by 5HT receptor antagonists from electrical stimulation of the median nerve [36]	***Rat study:*** 5-HT3 receptor antisense deoxynucleotides and $5HT_{1A}$ receptor antagonists failed to inhibit paracetamol antinociception [37]

**Nitric oxide synthase**	***Rat study:*** paracetamol inhibits substance P mediated hyperalgesia [38]<br><br>***Rat study:*** inhibitors of NOS produce antinociception and increase the analgesic activity of paracetamol [38]	***Rat study:*** Neither paracetamol nor its metabolite (4-acetamidophenol glucuronide) displayed any significant activity at constitutive NOS or inducible NOS [39]

Our understanding of the mechanistic effects of paracetamol is limited, but it remains widely used in the clinical setting. This is due to the adverse gastrointestinal, haemodynamic, cardiovascular and renal effects that accompany NSAID and opioid use [40]. Of particular relevance are the reduced bleeding complications with use of paracetamol.

As a result, current guidelines recommend paracetamol in the management of pain following many surgical procedures and in high-risk patients such as those with compromised livers, kidneys and those with coronary artery disease [4, 41, 42].

CLINICAL EFFECTS OF PARACETAMOL

1. Haemodynamic effects of IV paracetamol

IV paracetamol is widely used in the intensive care unit and for surgical patients. Recently, several studies have identified the incidence of hypotension with the use of IV paracetamol in critically ill patients. Maat et al., conducted haemodynamic studies on paracetamol following anecdotal accounts from ICU nurses of hypotension associated with IV paracetamol use [43]. Blood pressure measurements were taken prior to infusion and at 15 minute intervals for the first hour after administration and at 60 minute intervals until 4 hours after administration. Transient hypotension defined by a clinically relevant reduction in systolic blood pressure was observed in 22% of patients at the end of infusion (T=15 minutes) and in 33% of patients at 30 minutes after infusion ($p<0.003$). 16% of patients required intervention to correct blood pressure. Another study reported a reduction in systolic blood pressure by up to 13 mmHg ($p<0.0001$) and mean arterial pressure by 8 mmHg ($p<0.0001$) [44]. Additionally, in a study comparing effervescent paracetamol to normal oral paracetamol tablets, clinicians were warned of the potential for effervescent paracetamol to cause a rise blood pressure. The sodium content of effervescent paracetamol was implicated for the observed adverse haemodynamics [45]. It is therefore important to assess the effects of any additional compounds (e.g. mannitol) in the formulations of paracetamol to identify increased risk of adverse haemodynamic events.

Currently, the data has been largely gathered from small non-randomised, non-controlled observational studies that lack placebo groups therefore making it difficult to draw any direct conclusions regarding the haemodynamic effects of paracetamol. No studies have addressed the question as to whether these haemodynamic changes are due to:

- Any additional compounds such as mannitol or
- The antipyretic effect of paracetamol

The mechanism behind the haemodynamic changes observed in recent ICU studies after administration of IV paracetamol remains unknown however findings would be equally relevant to surgical patients in order to effectively manage the haemodynamic instability that accompanies the postoperative period.

2. Use of IV paracetamol in cardiac surgeries involving the cardiopulmonary bypass unit

Current guidelines have recommended paracetamol as a safe and efficacious analgesic for patients including undergoing high-risk surgeries including cardiac surgery [4, 41, 42].

Paracetamol has been advocated as a first line analgesic for patients with coronary artery disease on the assumption of its greater cardiovascular safety [4]. However, there have been few prospective studies on the effects of paracetamol on haemodynamics, pharmacokinetics, effective analgesia or antipyretic effects during and after cardiac surgery. Furthermore the effects of CPB on paracetamol levels are unknown. This is especially relevant since the consequence of haemodilution (which occurs during CPB) has a profound effect on blood pressure and the volume of distribution of administered drugs. In many instances, acute haemodilution leads to significant decreases in plasma drug concentrations and a permanent dilution of hydrophilic or non-protein bound compounds such as paracetamol [46]. Furthermore, significant amounts of drugs may sometimes be sequestered into the bypass equipment by incidental adsorption. Prophylactic doses that exceed the recommended maximum dosage for paracetamol may be important in maintaining a therapeutically effective concentration throughout the bypass procedure for post-operative analgesia.

It is therefore of clinical importance to understand the pharmacokinetic changes that may occur with CPB dependent cardiac surgeries, especially since the optimal route of administration and dose for paracetamol has not been extensively studied in this area.

3. Post-operative analgesic effect of IV paracetamol

Post-operatively, IV paracetamol administration is preferred over oral and rectal formulations. This is due to variable absorption of paracetamol in PO and PR use and longer time to achieve clinically relevant plasma concentrations (IV=15 minutes after infusion, oral = 1 hour after oral intake and rectal = variable depending on gastrointestinal motility of patient). IV paracetamol is considered preferred mode of administration for post-operative analgesic treatment.

The early intense pain following major surgeries, including cardiac surgery is complex. This is due to postoperative irritation and discomfort of:

- The endotracheal tube that is placed intraoperatively after general anaesthesia
- Wound drains (generally kept in place 24 hours post-operation)
- Pain at the incision site

The minimum concentration of paracetamol required for adequate post-operative analgesia is unknown. The 10 mg/ml required for therapeutic analgesia has been estimated from a paediatric tonsillectomy study [47] and therefore may not be accurate in adults or in different surgical patient subgroups such as cardiac surgical patients.

Postoperative pain is severe and complex. Paracetamol has been recommended as a safe analgesic for use in cardiac, liver and renal surgical patients. In randomised, controlled trials (RCT) on postoperative pain, paracetamol provided better analgesia compared to placebo group following dental, orthopaedic and gynaecological surgery [48-52]. However, opioid rescue was still necessary in these studies. Untreated postoperative pain secondary to cardiac surgery is a risk factor for cardiac complications [53]. As a result, untreated pain can increase heart rate and blood pressure.

Currently, only one study has observed the post-operative analgesic effects of cardiac surgery [54], however the investigators noted two major limitations:

1) Pain scores (VAS) were not taken before and after paracetamol administration, thus the acute analgesic effects of paracetamol were not investigated

2) The study failed to document the length of analgesic effect of paracetamol following cardiac surgery

It is therefore essential to include these factors in future studies to properly identify the acute analgesic effects of paracetamol.

The complexity of postoperative pain often requires multimodal analgesic management and treatment in conjunction with opioids has seen to be more effective [55]. The use of IV opioids is often indicated in post-operative pain, however the serious adverse effects associated with opioid has encouraged the use of alternative analgesics. While its exact mechanism is yet to be discovered, it is believed that paracetamol acts through different mechanisms in comparison to opioids, therefore the paracetamol-opioid combination is theorized to act synergistically in reducing pain [52]. A potential benefit of this multimodal technique is reduced opioid requirements for effective analgesia and decreased strength of opioid related side effects.

Currently, it is unclear as to whether IV paracetamol provides beneficial opioid-sparing effects with contradicting results from several studies, most of which used the pro-drug propacetamol. In one study propacetamol significantly reduced the need for alfentanil rescue following laproscopic surgery [56]. Lahtinen et al., however discovered propacetamol had negligible effects in enhancing opioid-based analgesia in coronary artery bypass graft patients during the first 72 hours after surgery. Additionally, it did not decrease the

requirements for opioid consumption and thus had no dilutional effects on opioid related side effects [57]. These results however do not reflect the decreased opioid consumption within the first 24 hours of the study in the propacetamol group. In contrast, Gehling [58] demonstrated an effective reduction in opioid requirements post-thyroidectomy and parathyroidectomy with the use of IV paracetamol. This was supported by similar findings by Petterson et al., who observed a 5mmHg reduction in the requirement for Ketobemidone (a morphine-like opioid) with IV paracetamol compared to oral paracetamol [59]. However, the clinical relevance of these punative, albeit statistically significant opioid sparing effects

remains contentious. Furthermore, Petterson's group found no statistical reduction in the incidence of opioid-related adverse events.

In summary, it has been demonstrated that the use of IV paracetamol reduces the amount of opioids required for effective analgesia in some surgical subgroups. Whether the opioid sparing effect of paracetamol is large enough to provide a clinically significant reduction in opoid-related adverse events requires further investigation. Several limitations to these studies have been reported including lack of placebo group and short durations of study periods which need to be considered in future studies.

4. <u>Antipyretic effect of IV paracetamol</u>

The intravenous mode of administration for paracetamol has increased its popularity in the intensive care unit and in cardiac surgical patients. IV paracetamol has a smaller time to latency compared with oral paracetamol and therefore can rapidly treat patients presenting with pyresis [14]. While the antipyretic efficacy in oral and rectal formulations has well been established, very few studies have examined the usefulness of IV paracetamol in different clinical settings. Only one placebo-controlled randomised trial has been conducted. Researchers at the University of Miami were able to validate the antipyretic efficacy of IV paracetamol [60], however this study was conducted on healthy volunteers rather than hospital patients. Study participants received RSE (reference standard endotoxin) treatment which mimics the effects of fever, in the absence of infection. Moreover, all participants were males with a mean age of 29.9 years and therefore cannot be representative of the greater community let alone critically ill patients. Additionally, IV paracetamol use is associated with the treatment of sepsis in the intensive care with or without fever. Selladurai et al. have suggested additional research to understand the reasons behind why paracetamol is given to febrile or non-febrile septic ICU patients [61]. The rationale for treatment with paracetamol in the intensive care unit needs to be addressed.

3. STUDY OBJECTIVES

3.1 HYPOTHESIS

Paracetamol (1g IV) has adverse effects on blood pressure in patients undergoing cardiac surgery.

<u>Primary endpoint:</u> Changes in systolic, diastolic and mean arterial pressure 15 minutes after infusion of paracetamol.

<u>Secondary endpoints:</u> Changes in cardiac output and cardiac index at the above time points, core body temperature, time to first post-operative request for opioid treatment, amount of opioid administered during stay in ICU, frequency of inotrope use, renal biomarkers (serum and urine NGAL and cystatin C) and pro-inflammatory effects (TNFα, IL6 and IL-10).

3.2 Study Aims

Primary aims:

- To identify any haemodynamic effects of IV paracetamol during and after cardiac surgery.
- To examine the effect the cardiopulmonary bypass on the pharmacokinetics of paracetamol.

Secondary aims:

- To identify any antipyretic effects of IV paracetamol during and after cardiac surgery.
- To identify whether IV paracetamol has an adequate analgesic effect and/or opioid sparing effect in the post-operative setting.

Sampling time points:

**1. Pre-infusion:**	Baseline
**2. Post infusion:**	5 minutes
	10 minutes
	15 minutes
	30 minutes
	Pre-cardiopulmonary bypass
	Rewarming
	Arrival in ICU
	6 hours after admission to ICU
	12 hours after admission to ICU

3.3 Outcome Measures

Primary outcome:
Haemodynamic changes measured by:
1) Systolic blood pressure
2) Diastolic blood pressure
3) Mean arterial pressure

Secondary outcomes:
- Cardiac output
- Cardiac index
- Plasma paracetamol levels
- Renal biomarkers including serum and urine NGAL, cystatin C
- Pro-inflammatory effects using TNFα, IL-6 and IL-10
- Core body temperature
- Time to first postoperative request for opioid treatment
- Amount of opioid administered during stay in ICU
- Frequency of inotrope use

Other data collected:

- Patient demographics including age, body mass index, gender
- Type of surgery
- Cardiopulmonary bypass time
- Aortic X-Clamp time
- Volume of cardiopulmonary bypass priming fluids
- Total volume of administered cardioplegia
- EuroSCORE and Parsonnet indices
- Duration of ICU stay
- Duration of hospital stay
- Adverse events including PONV (post-operative nausea and vomiting)

4. STUDY DESIGN

4.1 STUDY TYPE & DESIGN & SCHEDULE

A schematic overview of the study design is summarized in Figure 3.

This is a blinded, randomised, single-centre controlled clinical trial. Patients will be randomised to receive either intravenous paracetamol (1g/100mL) or control drug (100mL 0.9% normal saline) in a 1:1 fashion for perioperative care.

In the post-operative period, patients will remain allocated to their randomised study group and will receive 2 further infusions of the trial drugs 6- hourly post surgery.

Standard of care for all patients

All patients undergoing cardiac surgery at Austin Hospital are managed by surgical, anaesthesia and intensive care protocol designed to standardise patient care.

Standard monitoring for all patients undergoing cardiac surgery includes a 5-lead continuous electrocardiograph, pulse oximetry, capnography, urine output and core body temperature, invasive arterial monitoring and central venous pressure monitoring. Intra-operative normothermia is maintained with warm fluids and a forced-air warming device. A pulmonary artery catheter is routinely inserted for advanced haemodynamic monitoring in all patients as part of standard of care for all patients undergoing cardiac surgery in our institution. Evidence of adequate end-organ perfusion is guided by serum lactate, base deficit, ScVO2 >70, Bispectral index >40, and normal ST-segments.

Table 2. Concentrations of the major constituents in the study drugs

Constituent	Actavis IV Paracetamol (100mL)	0.9% Normal Saline (100mL)
**Paracetamol (g)**	1.0	0
**Mannitol (g)**	3.91	0
**Cysteine hydrochloride monohydrate (g)**	0.025	0
**Dibasic dehydrate (g)**	0.015	0
**Sodium (Na+) ions (mEq/L)**	0	150
**Chloride (Cl-) ions (mEq/L)**	0	150

Figure 3. Schematic overview of intraoperative study design

Participant consent in pre-admission anaesthesia clinic
N = 50

Inclusion criteria

- ✓ Adult patients > 18 years
- ✓ Elective cardiac surgery requiring cardiopulmonary bypass

Exclusion criteria

- Age less <18 years
- Pregnancy
- Chronic renal impairment (250µmol/L)
- BMI > 35kg/m^2
- Chronic liver disease
- Allergy to IV paracetamol
- Administration of paracetamol within 24 hours of data collection

RANDOMISATION

Control Group
N=25
Prior to commencement of surgery
To receive the standard surgical and anaesthesia care
\+
Bolus of 100mL 0.9% normal saline

Intravenous Paracetamol Group
N=25
Prior to commencement of surgery
To receive the standard surgical and anaesthesia care
\+
Bolus of 100mL (1g/100mL) Actavis IV Paracetamol

Intraoperatively: all patients to receive standard surgical and anaesthesia care

- ✓ ECG
- ✓ Arterial line
- ✓ Central venous catheter
- ✓ Pulmonary artery catheter
- ✓ Urinary catheter
- ✓ BIS monitor i.e. depth of anaesthesia
- ✓ Fluid warmer and bear hugger
- ✓ Fluid administration and anaesthesia as per routine care

Postoperative Care

- As per standard surgical protocol
- All patients to receive post-operative opioid and inotrope treatment as per standard protocols for patients undergoing cardiac surgery

Primary endpoint:

- ✓ Haemodynamic changes (systolic, diastolic and mean arterial blood pressure)

Secondary endpoints:

- ✓ Cardiac output
- ✓ Cardiac input
- ✓ Plasma paracetamol levels
- ✓ Renal biomarkers including serum and uring NGAL, cystatin C
- ✓ Pro-inflammatory effects using TNFα, IL-6 and IL-10
- ✓ Core body temperature
- ✓ Time to first postoperative request for opioid treatment
- ✓ Amount of opioid administered during stay in ICU
- ✓ Frequency of inotrope use

Other data collected: Patient demographics type of surgery, cardiopulmonary bypass time, total volume of cardiopulmonary bypass priming fluids and cardioplegia administered, EuroSCORE and Parsonnet indices, duration of ICU stay, duration of hospital stay, adverse events

Time points

PERIOPERATIVE:

- Baseline (BL)
- 5 minutes (T5)
- 10 minutes (T10)
- 15 minutes (T15)
- 30 minutes (T30)
- Pre-cardiopulmonary bypass
- Rewarming

POSTOPERATIVE:

- ICU arrival (ICU1)
- ICU 6 hours (ICU6)
- ICU 12 hours (ICU12)

Figure 4. Schematic overview of postoperative study design

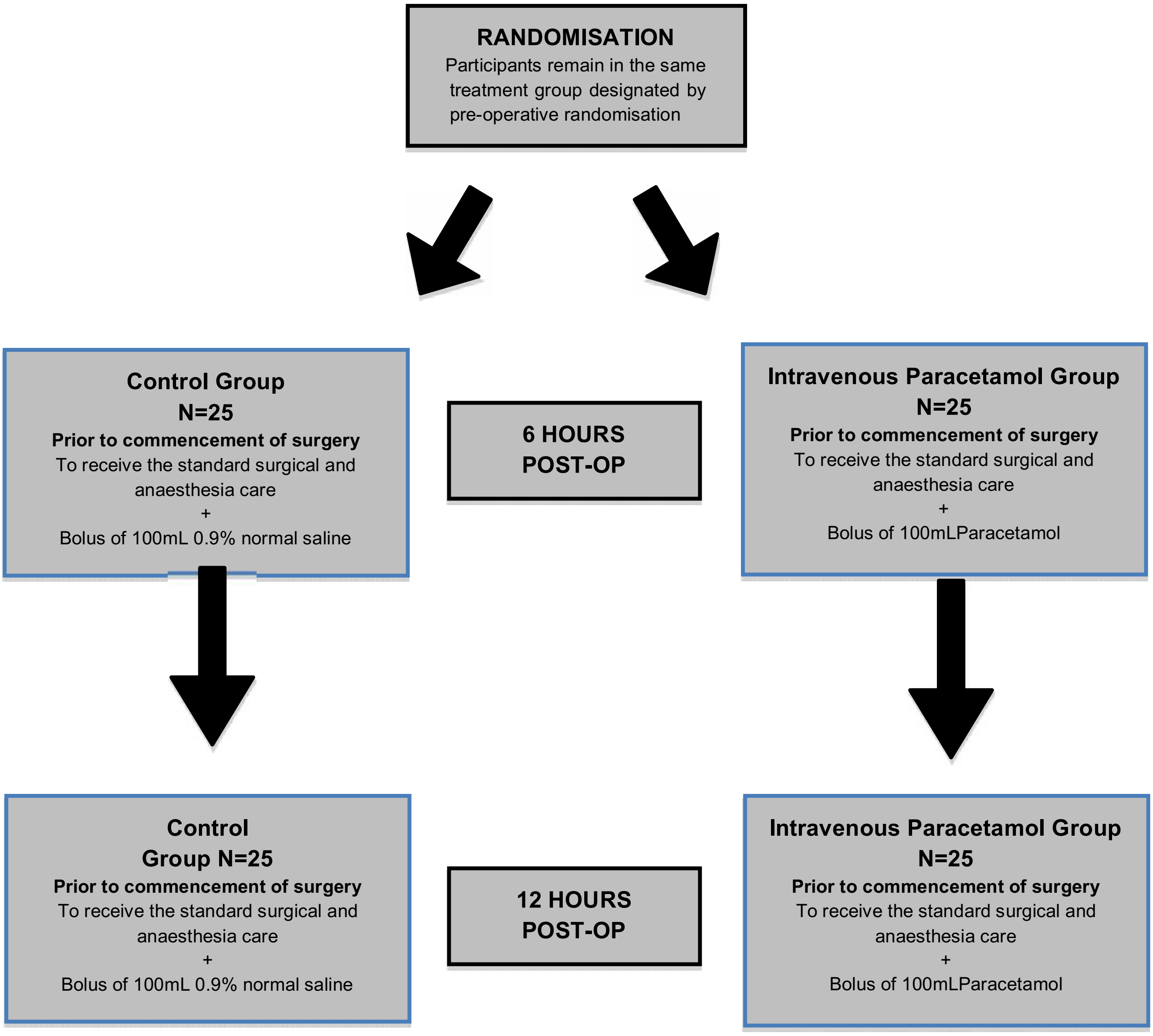

4.2 Standard Care and Additional to Standard Care Procedures

Standard Care Procedures		
**Procedure**	**Time/Visit**	**Dosage/ Volume**
Arterial line insertion	Intra-operatively	N/A
Central venous line insertion including pulmonary artery catheter	Intra-operatively	N/A

Additional To Standard Care		
**Procedure**	**Time/Visit**	**Dosage/ Volume**
Actavis IV paracetamol	***Pre-operative:*** Single dose ***Post-operative:*** Multiple doses 1/Q6H	1g/100mL
0.9% normal saline	***Pre-operative:*** Single dose ***Post-operative:*** Multiple doses 1/Q6H	100mL

4.3 Randomisation

Participants will be randomly assigned to one of two groups using a computer generated random number allocation system with permuted blocks prior to the commencement of the study.

Patients will be randomised to:

1) **Control group**: to receive 100mL 0.9% normal saline
2) **Intravenous paracetamol group**: to receive 1g/100mL Actavis IV paracetamol

Both groups will undergo cardiac surgery requiring cardiopulmonary bypass with fulfillment of the standard of care in our institution. In keeping with standard practices both groups will undergo preoperative multidisciplinary team assessment, where a cardiac surgeon, anaesthetist, and others will ensure that patients are optimized for their surgery.

In the perioperative period, participants will continue either the control treatment 6 hourly for 12 hours or paracetamol treatment 6 hourly for 12 hours (Figure 4).

Blinding

This is a blinded clinical trial. This study requires the use of 100mL blinded fluid vials. These vials will look identical in both patient groups and labeled as per Figure 5.

Figure 5. Labeling on blinded trial solution showing label information including assignment code located at the bottom right hand corner of the labels.

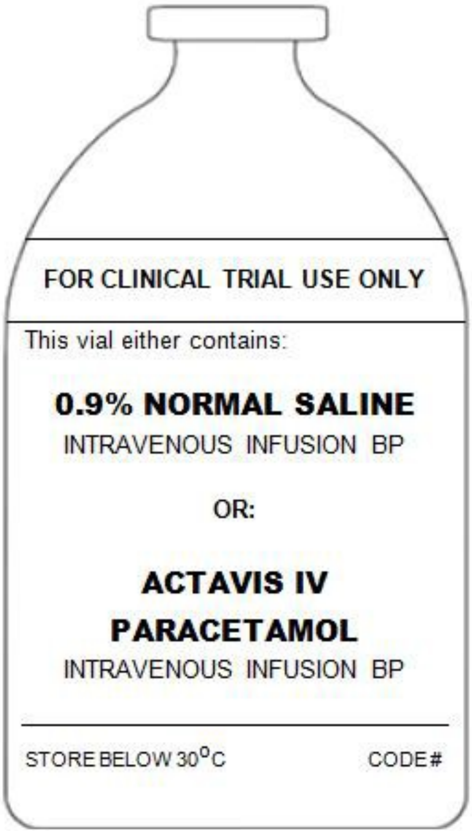

Prior to commencement of the study, each participant will be allocated an assignment code which is matched to the specific treatment group that the participant is randomised to. 25 participants will be allocated to one assignment code number and 25 patients will be allocated to the other. Depending on randomisation, 100mL IV paracetamol (1g/100mL) or 0.9% normal saline will be transferred into the blinded vials just prior to IV infusion by an independent ICU or anaesthesia research nurse. Importantly, study participants, and all clinicians and nursing personnel involved in their medical and surgical treatment will be blinded to treatment allocation.

SAFETY

In this study, in order to avoid any safety concerns with adverse haemodynamic effects: if a clinically significant decrease in blood pressure is observed (i.e. > 20% from baseline blood pressure), it will be managed as per conventional treatment for any hypotensive patient. The study drug will be immediately stopped and blood pressure will be normalized pharmacologically (e.g. Metaraminol bolus) or with intravenous fluids, in accordance with standard anaesthesia protocols for the management of intraoperative hypotension.

4.4 STUDY METHODOLOGY

Haemodynamic data collection

Data will be downloaded directly from theatre monitoring system to a designated computer at the end of the surgical case and in the intensive care unit. Time points for data collection are listed in the study table.

Urine samples for data collection

To study the effects of paracetamol on renal function, 5mL urine samples will be collected to calculate urine NGAL and cystatin C levels. Time points for data collection are listed in the study table.

Blood Samples for Data Collection:

7mL arterial blood samples will be taken at the following time points to calculate plasma-paracetamol concentrations, serum NGAL and pro-inflammatory effects. Time points for data collection are listed in the study table. All samples will be collected from the arterial line with the exception of one sample taken on rewarming at the end of CPB from the CPB machine itself.

Sampling time points: (✓ denotes sampling point)

	Assessment/ Procedure	BL	T1	T5	T10	T15	T30	Pre-CPB	Post-CPB	ICU1	ICU6	ICU12
Haemodynamic measurements	Systolic blood pressure	✓	✓	✓	✓	✓	✓	✓	✓	✓	✓	✓
	Diastolic blood pressure	✓	✓	✓	✓	✓	✓	✓	✓	✓	✓	✓
	Mean arterial pressure	✓	✓	✓	✓	✓	✓	✓	✓	✓	✓	✓
	Cardiac output	✓	✓	✓	✓	✓	✓	✓	✓	✓	✓	✓
	Cardiac index	✓	✓	✓	✓	✓	✓	✓	✓	✓	✓	✓
Biological samples and analysis	Blood sample (7mL)		✓			✓			✓			
	Plasma-PCML concentration		✓			✓			✓			
	TNFα analysis		✓			✓			✓			
	IL-6 analysis		✓			✓			✓			
	IL-10 analysis		✓			✓			✓			
	NGAL (serum)		✓			✓			✓			
	Urine sample (5mL)	✓								✓		
	NGAL (urine)	✓								✓		
Pyretic effect	Body temperature	✓	✓	✓	✓	✓	✓	✓	✓	✓	✓	✓
Analgesic effect	Time to first opioid use									During ICU Stay		
	Frequency of inotrope use									During ICU Stay		

PCML = paracetamol

BL = baseline (prior to administration of drug). **T1- 30** = time in minutes after infusion of drug

Pre-CPB = pre-cardiopulmonary bypass. Post-CPB = post-cardiopulmonary bypass

ICU1 = measurements taken 15 minutes after 1st infusion of paracetamol on arrival to ICU

ICU 6 and 12 = measurements taken 15 minutes after 2nd and 3rd infusion of paracetamol (6 hours and 12 hours respectively after arrival to ICU)

5. Study Population

5.1 Recruitment Procedure

All patients undergoing cardiac surgery with the requirement of cardiopulmonary bypass will be evaluated preoperatively at the anaesthesia pre-admissions clinic at least 1-2 weeks prior to surgery. Patients will be identified for study entry by the investigators or an anaesthetist or research co-ordinator acting on behalf of the principle investigators by surveillance of patients in the pre-admissions clinic. Patients will be identified from their preoperative medical records and surgical notes.

5.2 Inclusion Criteria

- All patients > 18 years
- Elective cardiac surgery requiring cardiopulmonary bypass

5.3 Exclusion Criteria

- Paracetamol use 24 hours prior to surgery (in paracetamol only or in combination therapy)
- Pregnancy
- Chronic renal impairment (250*u*mmol/L)
- Chronic liver disease (ALT > 200IU/L)
- Morbid obesity (BMI > 35kg/m^2)
- Known allergic reaction to IV paracetamol

5.4 Consent

A thorough assessment of the participant's competence and capacity to make a valid informed decision will be made by one of the study investigators prior to the patient being recruited. All patients will be deemed competent if they:

1. Are able to comprehend and retain information relevant to making the decision
2. Understand the information and implications of the decision
3. Are able to weigh the information in the balance and arrive at a decision

6. Participant Safety and Withdrawal

6.1 Risk Management and Safety

Recruitment for this study will in no way compromise standard of care for any patient undergoing this procedure. Both groups of patients will receive anaesthetic, perfusion and surgical treatment that is standard of care in our institution for all patients undergoing cardiac surgery involving the cardiopulmonary bypass machine.

All patients including the control group will have haemodynamic management (via inotropic use) guided by routine cardiovascular monitoring i.e. arterial line and pulmonary artery catheter measurements and post-operative pain management which will be at the discretion of the anesthetist, who will be blinded to the assignment of the patient.

6.2 Handling of Withdrawals

Participants may withdraw from the study at any point for the following reasons: participant has chosen to withdraw from the study or protocol violation. In these circumstances, the participants will be removed from analysis.

7. Statistical Methods

7.1 Sample Size Estimation & Justification

We will recruit 50 patients in total, 25 patients in the paracetamol group and 25 patients in the control group.

This is in keeping with realistic power calculations used in other studies evaluating the haemodynamic effects of IV paracetamol in patients undergoing major surgery.

7.2 Power Calculations

Sample size for the study was calculated based on our pilot data evaluating patients undergoing cardiac surgery at Austin hospital. With an average blood pressure of 120mmHg, and a SD of 10 mmHg, if we were to demonstrate a mean difference between the paracetamol group and control group of 10mmHg, with a power value of 80%, we require a minimum of 18 patients to be recruited into each group.

We will therefore recruit 25 patients in each arm, a total of 50 patients.

Summary of power calculations for this study

Confidence Interval (2-sided)		95%	
Power		80%	
Ratio of sample size (Group 2/Group 1)		1	
	**Group 1**		**Mean difference[1]**
Mean	120		10
Standard deviation	10		
Variance	0.0		

Sample size of Group 1	18
Sample size of Group 2	18
Total sample size	36

[1] Mean difference= (Group 1 mean) - (Group 2 mean)

RESULTS FROM OPENEPI, VERSION 2, OPEN SOURCE CALCULATOR--SSMEAN
http://www.openepi.com/OE2.3/SampleSize/SSMean.htm
Source file last modified on 09/21/2010 13:10:35

7.3 STATISTICAL METHODS TO BE UNDERTAKEN

Statistical analysis will be performed using computerized software (SPSS for Windows version 12.0). For data that was non-normally distributed a Mann–Whitney test was used and normally distributed data were compared using the Student T test.Ordinal and nominal data were compared using Chi square analysis. A p-value <0.05 will be considered significant.

8. Storage of Blood and Tissue Samples

8.1 Details of where samples will be stored, and the type of consent for future use of samples

All blood samples will be collected directly from the arterial line that will be inserted as standard of care. As the blood samples are taken directly from standard catheters while the patients are under general anaesthesia, **there will be no discomfort from the sampling of blood. Urine samples will be taken from a urinary catheter that is routinely inserted after general anaesthesia and will cause no additional discomfort on sampling**. Blood and urine samples will be transported in accordance with Austin Health's collection and transport protocols to the ICU where they will be centrifuged and stored in the -80°C freezer ready for bulk analysis. All samples will be analysed in accordance with Austin Health's laboratory processes and protocols. Only the tests described above (identical to the tests described in the patient information and consent form) will be performed on the blood samples in bulk batches at the end of the study. Under no circumstances will any other tests be performed. The privacy/confidentially of the samples will be maintained at all times

9. Data Security & Handling

9.1 Details of where records will be kept & How long will they be stored

The information to be stored during and after this RCT will be in the form of password protected electronic computer files on a dedicated hard drive in the department of anaesthesia. Data will be password protected on a dedicated storage file set up by the Information Technology department at Austin Hospital. Only the principle investigators will have access to this electronic database. Any paper data will be locked in dedicated research storage drawers in the department of anaesthesia.

All data records both electronic and paper will be retained for 15 years prior to destruction/shredding in accordance with Good Clinical Practice for clinical trials. Information will be disposed of securely by shredding of paper documents and permanent erasure of electronic data. Any back up in the form of CD discs or DVD's will be destroyed.

9.2 Confidentiality and Security

Data will be password protected on a dedicated storage file set up by the Information Technology department at Austin Hospital. Only the principle investigators will have access to this electronic database. Any paper data will be locked in dedicated research storage drawers in the department of anaesthesia.

Study organization

This study will be managed and coordinated by a team of clinician researchers with experience in the conduct of perioperative clinical trials. The Department of Intensive Care Research Group will centrally manage the project.

Detailed Budget

Item	Year 1
**Cost of intravenous paracetamol**<br>100mL Actavis IV paracetamol:<br>Cost: $1.00 per unit	$25
**Cost of 0.9% normal saline**<br>100mL 0.9% normal saline<br>Cost: $0.50 per unit	$12.50
**Cost of Paracetamol-Plasma assay:**<br>5 assays per patient: $15 per patient<br>Total of 150 samples	$2250
**Cost of Interleukin-6 assay: $40**<br>5 assays per patient: $40 per patient<br>Total of 150 samples	$4000
**Cost of Interleukin-10 assay: $40**<br>5 assays per patient: $40 per patient<br>Total of 150 samples	$4000
**Cost of TNFα assay: $40**<br>5 assays per patient: $40 per patient<br>Total of 150 samples	$4000
**Urinary and plasma NGAL assay:**<br>5 assays per patient: $40 patient:<br>Total of 250 samples	$10000

Maintenance

Item	Year 1
Computer consumables (printing, paper, ink)	500
Professional statistical consultation	2,000

Other Items

Item	Year 1
Fee to Human Research Ethics Committees: Investigator initiated	$200

**Total (AUD)**	**$30987.50**

Timelines and milestones

Date	Project Milestone
March 2013	Ethics application and subsequent regulatory requirements
May 2013	Commence recruitment
May 2014	Database lock, data analysis and initial results
Dec 2014	Publication

10. APPENDIX

List of Attachments included:

Document Name	Version Number	Date
Actavis IV Paracetamol Product Information Sheet	1	20 June 2011
Normal Saline 0.9% Product Information Sheet	1	9 October 2002

11. REFERENCES

1. Wang, J.H., et al., *Pulmonary edema in the transurethral resection syndrome induced with mannitol 5%.* Acta Anaesthesiol Scand, 2009. **53**(8): p. 1094-6.
2. Krenzelok, E.P. and M.A. Royal, *Confusion: acetaminophen dosing changes based on NO evidence in adults.* Drugs R D, 2012. **12**(2): p. 45-8.
3. IMS, *IMS Institute for Healthcare Informations: The Use of Medicines in the United States*, 2011.
4. Sudano, I., et al., *Acetaminophen increases blood pressure in patients with coronary artery disease.* Circulation, 2010. **122**(18): p. 1789-96.
5. Radack, K.L., C.C. Deck, and S.S. Bloomfield, *Ibuprofen interferes with the efficacy of antihypertensive drugs. A randomized, double-blind, placebo-controlled trial of ibuprofen compared with acetaminophen.* Ann Intern Med, 1987. **107**(5): p. 628-35.
6. Chua, S.S., et al., *Cardiovascular effects of a chlorpheniramine/paracetamol combination in hypertensive patients who were sensitive to the pressor effect of pseudoephedrine.* Br J Clin Pharmacol, 1991. **31**(3): p. 360-2.
7. Pavlicevic, I., et al., *Interaction between antihypertensives and NSAIDs in primary care: a controlled trial.* Can J Clin Pharmacol, 2008. **15**(3): p. e372-82.
8. Boyle, M., S. Hundy, and T.A. Torda, *Paracetamol administration is associated with hypotension in the critically ill.* Aust Crit Care, 1997. **10**(4): p. 120-2.
9. Krajcova, A., V. Matousek, and F. Duska, *Mechanism of paracetamol-induced hypotension in critically ill patients: A prospective observational cross-over study.* Aust Crit Care, 2012.
10. Duggan, S.T. and L.J. Scott, *Intravenous paracetamol (acetaminophen).* Drugs, 2009. **69**(1): p. 101-13.
11. Holmér Pettersson, P., et al., *Early bioavailability in day surgery: a comparison between orally, rectally, and intravenously administered paracetamol.* Ambulatory Surgery, 2005. **12**(1): p. 27-30.
12. Cullen, S., et al., *Paracetamol suppositories: a comparative study.* Arch Dis Child, 1989. **64**(10): p. 1504-5.
13. Anderson, B.J., J. Monteleone, and N.H. Holford, *Variability of concentrations after rectal paracetamol.* Paediatr Anaesth, 1998. **8**(3): p. 274.
14. Singla, N.K., et al., *Plasma and cerebrospinal fluid pharmacokinetic parameters after single-dose administration of intravenous, oral, or rectal acetaminophen.* Pain Pract, 2012. **12**(7): p. 523-32.
15. Berl, V., A. Barbaud, and J.P. Lepoittevin, *Mechanism of allergic contact dermatitis from propacetamol: sensitization to activated N,N-diethylglycine.* Contact Dermatitis, 1998. **38**(4): p. 185-8.
16. Murat, I., et al., *Tolerance and analgesic efficacy of a new i.v. paracetamol solution in children after inguinal hernia repair.* Paediatr Anaesth, 2005. **15**(8): p. 663-70.
17. McNicol, E.D., et al., *Single-dose intravenous paracetamol or propacetamol for prevention or treatment of postoperative pain: a systematic review and meta-analysis.* Br J Anaesth, 2011. **106**(6): p. 764-75.
18. Vane, J.R. and R.M. Botting, *Mechanism of action of nonsteroidal anti-inflammatory drugs.* Am J Med, 1998. **104**(3A): p. 2S-8S; discussion 21S-22S.
19. Cashman, J. and G. McAnulty, *Nonsteroidal anti-inflammatory drugs in perisurgical pain management. Mechanisms of action and rationale for optimum use.* Drugs, 1995. **49**(1): p. 51-70.
20. Cashman, J.N., *The mechanisms of action of NSAIDs in analgesia.* Drugs, 1996. **52 Suppl 5**: p. 13-23.
21. Becker, J.C., W. Domschke, and T. Pohle, *Current approaches to prevent NSAID-induced gastropathy--COX selectivity and beyond.* Br J Clin Pharmacol, 2004. **58**(6): p.587-600.

22. Mattia, A. and F. Coluzzi, *What anesthesiologists should know about paracetamol (acetaminophen).* Minerva Anestesiol, 2009. **75**(11): p. 644-53.
23. Ayoub, S.S., et al., *The involvement of a cyclooxygenase 1 gene-derived protein in the antinociceptive action of paracetamol in mice.* Eur J Pharmacol, 2006. **538**(1-3): p. 57-65.
24. Li, S., et al., *Acetaminophen: antipyretic or hypothermic in mice? In either case, PGHS-1b (COX-3) is irrelevant.* Prostaglandins Other Lipid Mediat, 2008. **85**(3-4): p. 89-99.
25. Flower, R.J. and J.R. Vane, *Inhibition of prostaglandin synthetase in brain explains the anti-pyretic activity of paracetamol (4-acetamidophenol).* Nature, 1972. **240**(5381): p. 410-1.
26. Botting, R. and S.S. Ayoub, *COX-3 and the mechanism of action of paracetamol/acetaminophen.* Prostaglandins Leukot Essent Fatty Acids, 2005. **72**(2): p. 85-7.
27. Snipes, J.A., et al., *Cloning and characterization of cyclooxygenase-1b (putative cyclooxygenase-3) in rat.* J Pharmacol Exp Ther, 2005. **313**(2): p. 668-76.
28. Toussaint, K., et al., *What do we (not) know about how paracetamol (acetaminophen) works?* J Clin Pharm Ther, 2010. **35**(6): p. 617-38.
29. Warner, T.D. and J.A. Mitchell, *Cyclooxygenase-3 (COX-3): filling in the gaps toward a COX continuum?* Proc Natl Acad Sci U S A, 2002. **99**(21): p. 13371-3.
30. Botting, R.M., *Mechanism of action of acetaminophen: is there a cyclooxygenase 3?* Clin Infect Dis, 2000. **31 Suppl 5**: p. S202-10.
31. Szallasi, A. and V. Di Marzo, *New perspectives on enigmatic vanilloid receptors.* Trends Neurosci, 2000. **23**(10): p. 491-7.
32. Bertolini, A., et al., *Paracetamol: new vistas of an old drug.* CNS Drug Rev, 2006. **12**(3-4): p. 250-75.
33. Abbott, F.V. and K.G. Hellemans, *Phenacetin, acetaminophen and dipyrone: analgesic and rewarding effects.* Behav Brain Res, 2000. **112**(1-2): p. 177-86.
34. Srikiatkhachorn, A., N. Tarasub, and P. Govitrapong, *Acetaminophen-induced antinociception via central 5-HT(2A) receptors.* Neurochem Int, 1999. **34**(6): p. 491-8.
35. Courade, J.P., et al., *5-HT receptor subtypes involved in the spinal antinociceptive effect of acetaminophen in rats.* Eur J Pharmacol, 2001. **432**(1): p. 1-7.
36. Pickering, G., et al., *Analgesic effect of acetaminophen in humans: first evidence of a central serotonergic mechanism.* Clin Pharmacol Ther, 2006. **79**(4): p. 371-8.
37. Libert, F., et al., *Acetaminophen: a central analgesic drug that involves a spinal tropisetron-sensitive, non-5-HT(3) receptor-mediated effect.* Mol Pharmacol, 2004. **66**(3): p. 728-34.
38. Bujalska, M., *Effect of nitric oxide synthase inhibition on antinociceptive action of different doses of acetaminophen.* Pol J Pharmacol, 2004. **56**(5): p. 605-10.
39. Raffa, R.B., *Acetaminophen and metabolite: lack of effect on nitric oxide synthase (constitutive or inducible).* Headache, 2002. **42**(3): p. 237-8.
40. Maund, E., et al., *Paracetamol and selective and non-selective non-steroidal anti-inflammatory drugs for the reduction in morphine-related side-effects after major surgery: a systematic review.* Br J Anaesth, 2011. **106**(3): p. 292-7.
41. Galinski, M., et al., *Reduction of paracetamol metabolism after hepatic resection.* Pharmacology, 2006. **77**(4): p. 161-5.
42. Morgan, S., *Intravenous paracetamol in patients with renal colic.* Emerg Nurse, 2011. **18**(9): p. 22-5.
43. de Maat, M.M., et al., *Paracetamol for intravenous use in medium--and intensive care patients: pharmacokinetics and tolerance.* Eur J Clin Pharmacol, 2010. **66**(7): p. 713-9.
44. Duncan, C.N., J. Seet, and S. Baker, *Centrally administered parenteral paracetamol: A potentially under-reported cause of haemodynamic instability within the adult intensive care unit.* Australian Critical Care, 2012. **25**(2): p. 131.
45. Ubeda, A., J. Llopico, and M.T. Sanchez, *Blood pressure reduction in hypertensive patients after withdrawal of effervescent medication.* Pharmacoepidemiol Drug Saf, 2009. **18**(5): p. 417-9.

46. Pea, F., F. Pavan, and M. Furlanut, *Clinical relevance of pharmacokinetics and pharmacodynamics in cardiac critical care patients.* Clin Pharmacokinet, 2008. **47**(7): p. 449-62.
47. Anderson, B., S. Kanagasundarum, and G. Woollard, *Analgesic efficacy of paracetamol in children using tonsillectomy as a pain model.* Anaesth Intensive Care, 1996. **24**(6): p. 669-73.
48. Sinatra, R.S., et al., *Efficacy and safety of single and repeated administration of 1 gram intravenous acetaminophen injection (paracetamol) for pain management after major orthopedic surgery.* Anesthesiology, 2005. **102**(4): p. 822-31.
49. Candiotti K, S.N., Wininger S, et al., *A randomized, double-blind, placebo-controlled, multi-center, parallel-group, multiple-dose study of the efficacy and safety of intravenous acetaminophen*

over 48 hours for the treatment of postoperative pain after gynecologic surgery [abstract no. A-13 plus poster]. 33rd Annual Regional Anesthesia Meeting, 2008. **May 1-4; Cancun**.
50. Juhl, G.I., et al., *Analgesic efficacy and safety of intravenous paracetamol (acetaminophen) administered as a 2 g starting dose following third molar surgery.* Eur J Pain, 2006. **10**(4): p. 371-7.
51. Moller, P.L., et al., *Intravenous acetaminophen (paracetamol): comparable analgesic efficacy, but better local safety than its prodrug, propacetamol, for postoperative pain after third molar surgery.* Anesth Analg, 2005. **101**(1): p. 90-6, table of contents.
52. D, G., *Principles of Pharmacology*. 1st Edition ed. 2005: Lippincott Williams & Wilkins.
53. Roediger, L., R. Larbuisson, and M. Lamy, *New approaches and old controversies to postoperative pain control following cardiac surgery.* Eur J Anaesthesiol, 2006. **23**(7): p. 539-50.
54. Cattabriga, I., et al., *Intravenous paracetamol as adjunctive treatment for postoperative pain after cardiac surgery: a double blind randomized controlled trial.* Eur J Cardiothorac Surg, 2007. **32**(3): p. 527-31.
55. Buvanendran, A. and J.S. Kroin, *Multimodal analgesia for controlling acute postoperative pain.* Curr Opin Anaesthesiol, 2009. **22**(5): p. 588-93.
56. Hahn, T.W., et al., *Analgesic effect of i.v. paracetamol: possible ceiling effect of paracetamol in postoperative pain.* Acta Anaesthesiol Scand, 2003. **47**(2): p. 138-45.
57. Lahtinen, P., et al., *Propacetamol as adjunctive treatment for postoperative pain after cardiac surgery.* Anesth Analg, 2002. **95**(4): p. 813-9, table of contents.
58. Gehling, M., et al., *Postoperative analgesia with parecoxib, acetaminophen, and the combination of both: a randomized, double-blind, placebo-controlled trial in patients undergoing thyroid surgery.* Br J Anaesth, 2010. **104**(6): p. 761-7.
59. Pettersson, P.H., J. Jakobsson, and A. Owall, *Intravenous acetaminophen reduced the use of opioids compared with oral administration after coronary artery bypass grafting.* J Cardiothorac Vasc Anesth, 2005. **19**(3): p. 306-9.
60. Kett, D.H., et al., *A randomized study of the efficacy and safety of intravenous acetaminophen vs. intravenous placebo for the treatment of fever.* Clin Pharmacol Ther, 2011. **90**(1): p. 32-9.
61. Selladurai, S., et al., *Paracetamol therapy for septic critically ill patients: a retrospective observational study.* Crit Care Resusc, 2011. **13**(3): p. 181-6.
