# Supplementary material for: The hemodynamic effects of intravenous paracetamol (acetaminophen) vs normal saline in cardiac surgery patients: A single center placebo controlled randomized study
Source: PLoS One. 2018 Apr 16;13(4):e0195931. doi: 10.1371/journal.pone.0195931 (PMC5901786; doi:10.1371/journal.pone.0195931)
Supplement: S1 Table — Data analysed using a random-effect generalized least squares regression model. (DOCX) [file pone.0195931.s004.docx]

**Supporting information Table 4: Longitudinal data of endpoints during and after a 15-minute infusion of intravenous paracetamol administered preoperatively. Data analysed using a random-effect generalized least squares (GLS) regression model. Values are mean values (standard deviation) and confidence intervals (95% CI).**

|  |  | **Baseline** | **5 mins** | **8 mins** | **10 mins** | **15 mins** | **30 mins** | **Mean difference**  **(Estimated from GLS model)** | **95% CI**  **(Estimated from GLS model)** | ***p* value (treatment)** | ***p* value (treatment-by-time interaction)** |
| --- | --- | --- | --- | --- | --- | --- | --- | --- | --- | --- | --- |
| SBP (mmHg) | **Paracetamol** | 136 (21) | 130 (28) | 127 (24) | 129 (26) | 126 (27) | 124 (26) | 0.75 | -12 to 14 | 0.91 | 0.002 |
|  | **Saline** | 130 (24) | 126 (22) | 128 (22) | 128 (23) | 126 (23) | 129 (23) |  |  |  |  |
| DBP (mmHg) | **Paracetamol** | 67 (10) | 63 (13) | 62 (12) | 61 (11) | 60 (12) | 61 (14) | 2.14 | -4 to 8 | 0.47 | <0.001 |
|  | **Saline** | 61 (10) | 60 (12) | 60 (11) | 60 (11) | 58 (10) | 59 (16) |  |  |  |  |
| MAP (mmHg) | **Paracetamol** | 90 (12) | 83 (21) | 85 (15) | 85 (15) | 84 (16) | 82 (14) | 0.19 | -8 to 8 | 0.96 | 0.15 |
|  | **Saline** | 87 (17) | 83 (16) | 85 (15) | 85 (16) | 83 (15) | 86 (17) |  |  |  |  |
| sPAP (mmHg) | **Paracetamol** | 27 (9) | 28 (11) | 29 (11) | 29 (10) | 30 (10) | 29 (11) | -2.49 | -8 to 4 | 0.42 | 0.88 |
|  | **Saline** | 31 (11) | 30 (13) | 31 (13) | 32 (12) | 32 (11) | 32 (13) |  |  |  |  |
| dPAP (mmHg) | **Paracetamol** | 15 (6) | 14 (7) | 15 (7) | 14 (6) | 16 (7) | 15 (7) | 0.18 | -3 to 4 | 0.92 | 0.94 |
|  | **Saline** | 14 (7) | 14 (9) | 15 (8) | 15 (7) | 15 (7) | 15 (8) |  |  |  |  |
| mPAP (mmHg) | **Paracetamol** | 19 (8) | 20 (8) | 21 (9) | 21 (7) | 22 (8) | 21 (8) | -0.58 | -5 to 3 | 0.80 | 0.94 |
|  | **Saline** | 23 (13) | 20 (10) | 21 (10) | 21 (9) | 22 (9) | 22 (10) |  |  |  |  |
| CVP (mmHg) | **Paracetamol** | 9 (5) | 7 (5) | 8 (5) | 7 (4) | 9 (6) | 8 (5) | -0.49 | -3 to 2 | 0.74 | 0.12 |
|  | **Saline** | 7 (5) | 9 (7) | 9 (7) | 9 (6) | 9 (7) | 8 (6) |  |  |  |  |
| HR (beats/min) | **Paracetamol** | 72 (13) | 71 (13) | 72 (13) | 70 (13) | 71 (13) | 71 (13) | 6.94 | 0.40 to 13 | 0.04 | 0.4 |
|  | **Saline** | 66 (12) | 64 (11) | 64 (11) | 64 (11) | 65 (11) | 64 (12) |  |  |  |  |
| CI (L min**^-1^** m^2^) | **Paracetamol** | 2.72 (1.02) | not measured | 2.72 (0.96) | not measured | 2.58 (0.68) | 2.72 (0.79) | 0.27 | -0.10 to 0.64 | 0.15 | 0.92 |
|  | **Saline** | 2.49 (1.00) | not measured | 2.36 (0.57) | not measured | 2.45 (0.56) | 2.49 (0.64) |  |  |  |  |
| SVRI (dynes sec**^-1^** cm**^-^**^5^ m^2^) | **Paracetamol** | 2695 (1027) | not measured | 2476 (805) | not measured | 2459 (680) | 2363 (911) | -137 | -508 to 233 | 0.47 | 0.68 |
|  | **Saline** | 2746 (680) | not measured | 2678 (580) | not measured | 2501 (615) | 2580 (629) |  |  |  |  |
