# Supplementary material for: The hemodynamic effects of intravenous paracetamol (acetaminophen) vs normal saline in cardiac surgery patients: A single center placebo controlled randomized study
Source: PLoS One. 2018 Apr 16;13(4):e0195931. doi: 10.1371/journal.pone.0195931 (PMC5901786; doi:10.1371/journal.pone.0195931)
Supplement: S2 Table — Data analysed using a random-effect generalized least squares regression model. Changes in postoperative. (DOCX) [file pone.0195931.s005.docx]

|  |  | **Baseline** | **15 mins** | **30 mins** | **1 hr** | **2 hr** | **3 hr** | **4 hr** | **5 hr** | **6 hr** | **Mean difference**  **(Estimated from GLS model)** | **95% CI**  **(estimated from GLS model)** | ***p* value**  **(treatment)** | ***p* value**  **(treatment-by-time interaction)** |
| --- | --- | --- | --- | --- | --- | --- | --- | --- | --- | --- | --- | --- | --- | --- |
| SBP (mmHg) | **Paracetamol** | 103 (11) | 103 (11) | 107 (16) | 102 (11) | 104 (13) | 110 (21) | 108 (13) | 112 (12) | 109 (11) | -9.99 | -22 to 2 | 0.1 | 0.006 |
|  | **Saline** | 115 (21) | 117 (26) | 113 (25) | 116 (22) | 157 (27) | 106 (20) | 107 (18) | 105 (16) | 108 (12) |  |  |  |  |
| DBP (mmHg) | **Paracetamol** | 56 (9) | 56 (9) | 57 (9) | 55 (8) | 55 (8) | 59 (12) | 57 (10) | 59 (10) | 57 (10) | -0.43 | -6 to 5 | 0.87 | 0.018 |
|  | **Saline** | 61 (18) | 60 (17) | 60 (18) | 59 (14) | 58 (11) | 55 (10) | 56 (10) | 54 (10) | 58 (12) |  |  |  |  |
| MAP (mmHg) | **Paracetamol** | 72 (9) | 72 (9) | 74 (11) | 71 (8) | 72 (8) | 76 (13) | 75 (9) | 77 (9) | 75 (9) | -0.15 | -5 to 5 | 0.96 | 0.006 |
|  | **Saline** | 79 (17) | 79 (21) | 76 (23) | 78 (18) | 76 (14) | 76 (22) | 73 (12) | 71 (12) | 74 (11) |  |  |  |  |
| sPAP (mmHg) | **Paracetamol** | 35 (11) | 35 (10) | 37 (11) | 36 (10) | 37 (10) | 39 (12) | 37 (11) | 37 (10) | 35 (10) | 0.75 | -4 to 5 | 0.75 | 0.53 |
|  | **Saline** | 35 (9) | 35 (10) | 35 (10) | 35 (7) | 36 (7) | 37 (7) | 38 (6) | 35 (8) | 35 (7) |  |  |  |  |
| dPAP (mmHg) | **Paracetamol** | 18 (5) | 18 (5) | 18 (6) | 18 (5) | 18 (5) | 20 (6) | 19 (5) | 18 (5) | 17 (5) | 0.53 | -2 to 3 | 0.64 | 0.74 |
|  | **Saline** | 18 (7) | 18 (7) | 18 (6) | 18 (5) | 18 (4) | 18 (4) | 18 (3) | 17 (4) | 17 (4) |  |  |  |  |
| mPAP (mmHg) | **Paracetamol** | 24 (7) | 24 (6) | 25 (7) | 25 (6) | 25 (6) | 27 (8) | 26 (7) | 26 (6) | 24 (7) | 0.76 | -2 to 4 | 0.59 | 0.69 |
|  | **Saline** | 24 (7) | 25 (7) | 24 (7) | 25 (5) | 24 (7) | 25 (4) | 25 (4) | 24 (5) | 24 (4) |  |  |  |  |
| CVP (mmHg) | **Paracetamol** | 12 (4) | 13 (6) | 13 (8) | 13 (4) | 12 (4) | 14 (5) | 13 (4) | 13 (4) | 12 (4) | 0.36 | -1 to 2 | 0.7 | 0.33 |
|  | **Saline** | 13 (5) | 12 (5) | 13 (5) | 12 (5) | 12 (4) | 13 (4) | 12 (4) | 11 (5) | 13 (3) |  |  |  |  |
| HR (beats/min) | **Paracetamol** | 90 (12) | 90 (12) | 91 (11) | 90 (11) | 90 (14) | 91 (16) | 91 (14) | 91 (13) | 90 (14) | 2.23 | -3 to 8 | 0.43 | 0.69 |
|  | **Saline** | 88 (8) | 87 (8) | 87 (9) | 87 (7) | 90 (8) | 89 (9) | 89 (10) | 88 (9) | 87 (8) |  |  |  |  |
| CI (L min^-1^ m^2^) | **Paracetamol** | 2.49 (0.48) | 2.58 (0.56) | 2.68 (0.52) | 2.59 (0.44) | 2.67 (0.60) | 2.73 (0.59) | 2.79 (0.73) | 2.77 (0.64) | 2.78 (0.61) | -0.07 | -0.3 to 0.18 | 0.57 | 0.005 |
|  | **Saline** | 2.57 (0.58) | 2.54 (0.55) | 2.57 (0.57) | 2.65 (0.58) | 2.69 (0.53) | 2.81 (0.48) | 2.89 (0.64) | 3.09 (0.98) | 2.92 (0.79) |  |  |  |  |
| SVRI (dynes sec^-1^ cm^-5^ m^2^) | **Paracetamol** | 2016 (496) | 1911 (528) | 1895 (599) | 1860 (431) | 1858 (433) | 1891 (521) | 1878 (511) | 1967 (502) | 1920 (460) | 0.64 | -114 to 162 | 0.72 | 0.72 |
|  | **Saline** | 2169 (718) | 2280 (1186) | 2103 (1001) | 2118 (803) | 1974 (624) | 1556 (785) | 1766 (522) | 1688 (628) | 1836 (636) |  |  |  |  |

**Supplementary Table 5: Longitudinal data of endpoints after intravenous paracetamol administered immediately after cardiac surgery. Data analysed using a random-effect generalized least squares (GLS) regression model. Values are mean values (standard deviation) and confidence intervals (95% CI).**
