# Supplementary material for: The hemodynamic effects of intravenous paracetamol (acetaminophen) vs normal saline in cardiac surgery patients: A single center placebo controlled randomized study
Source: PLoS One. 2018 Apr 16;13(4):e0195931. doi: 10.1371/journal.pone.0195931 (PMC5901786; doi:10.1371/journal.pone.0195931)
Supplement: S3 Table — Data analysed using random-effect generalized least squares regression model. (DOCX) [file pone.0195931.s006.docx]

**Supplementary Table 6: Longitudinal data of endpoints after intravenous paracetamol administered 6 hours after cardiac surgery. Data analysed using random-effect generalized least squares (GLS) regression model. Values are mean values (standard deviation) and confidence intervals (95% CI).**

|  |  | **Baseline** | **15 mins** | **30 mins** | **1 hr** | **2 hr** | **3 hr** | **4 hr** | **5 hr** | **6 hr** | **Mean difference**  **(Estimated from GLS model)** | **95% CI**  **(Estimated from GLS model)** | ***p* value (treatment)** | ***p* value (treatment-by-time interaction)** |
| --- | --- | --- | --- | --- | --- | --- | --- | --- | --- | --- | --- | --- | --- | --- |
| SBP (mmHg) | **Paracetamol** | 109 (11) | 108 (11) | 107 (11) | 109 (13) | 111 (11) | 112 (13) | 113 (15) | 115 (14) | 117 (16) | 3.26 | -7 to 13 | 0.53 | 0.15 |
|  | **Saline** | 109 (12) | 108 (17) | 109 (17) | 110 (17) | 111 (15) | 114 (13) | 114 (15) | 114 (19) | 117 (21) |  |  |  |  |
| DBP (mmHg) | **Paracetamol** | 57 (10) | 57 (10) | 57 (9) | 57 (10) | 57 (10) | 59 (11) | 57 (11) | 57 (9) | 58 (10) | 1.37 | -3 to 6 | 0.57 | 0.4 |
|  | **Saline** | 58 (12) | 56 (10) | 56 (9) | 57 (9) | 56 (9) | 57 (7) | 56 (9) | 55 (10) | 56 (9) |  |  |  |  |
| MAP (mmHg) | **Paracetamol** | 75 (9) | 75 (9) | 75 (8) | 76 (9) | 76 (8) | 78 (9) | 77 (10) | 77 (9) | 78 (8) | 2.12 | -2 to 7 | 0.36 | 0.025 |
|  | **Saline** | 75 (11) | 74 (12) | 73 (11) | 75 (11) | 74 (12) | 75 (8) | 74 (9) | 74 (11) | 76 (11) |  |  |  |  |
| sPAP (mmHg) | **Paracetamol** | 35 (10) | 35 (10) | 35 (10) | 35 (11) | 34 (10) | 35 (10) | 36 (11) | 35 (10) | 36 (10) | -0.37 | -5 to 4 | 0.87 | 0.59 |
|  | **Saline** | 35 (7) | 33 (7) | 34 (5) | 35 (7) | 35 (8) | 36 (10) | 36 (9) | 36 (9) | 36 (10) |  |  |  |  |
| dPAP (mmHg) | **Paracetamol** | 17 (5) | 17 (5) | 17 (5) | 17 (6) | 16 (5) | 17 (5) | 17 (6) | 16 (5) | 17 (5) | -0.18 | -2 to 2 | 0.87 | 0.1 |
|  | **Saline** | 17 (4) | 16 (4) | 17 (3) | 18 (4) | 17 (5) | 17 (5) | 15 (4) | 16 (4) | 17 (4) |  |  |  |  |
| mPAP (mmHg) | **Paracetamol** | 24 (6) | 24 (6) | 24 (6) | 24 (7) | 23 (6) | 24 (6) | 24 (7) | 24 (6) | 24 (6) | -0.2 | -3 to 3 | 0.88 | 0.66 |
|  | **Saline** | 24 (4) | 22 (6) | 23 (3) | 24 (4) | 24 (6) | 24 (6) | 24 (5) | 23 (5) | 24 (6) |  |  |  |  |
| CVP (mmHg) | **Paracetamol** | 12 (4) | 13 (3) | 14 (8) | 12 (4) | 11 (5) | 12 (4) | 12 (5) | 13 (6) | 12 (4) | 0.03 | -2 to 2 | 0.98 | 0.99 |
|  | **Saline** | 13 (3) | 12 (4) | 12 (3) | 12 (4) | 12 (4) | 12 (6) | 11 (4) | 12 (4) | 13 (4) |  |  |  |  |
| HR (beats/min) | **Paracetamol** | 90 (14) | 89 (15) | 88 (13) | 88 (12) | 87 (13) | 87 (13) | 87 (13) | 87 (13) | 87 (13) | 1.7 | -4 to 7 | 0.55 | 0.1 |
|  | **Saline** | 88 (8) | 87 (7) | 88 (8) | 87 (8) | 88 (12) | 86 (8) | 85 (8) | 84 (8) | 83 (7) |  |  |  |  |
| CI (L min^-1^ m^2^) | **Paracetamol** | 2.76 (0.62) | 2.77 (0.55) | 2.82 (0.63) | 2.88 (0.62) | 2.83 (0.60) | 2.84 (0.62) | 2.85 (0.68) | 2.79 (0.64) | 2.75 (0.49) | -0.03 | -0.33 to 0.26 | 0.81 | 0.36 |
|  | **Saline** | 2.91 (0.80) | 2.90 (0.72) | 2.97 (0.68) | 0.99 (0.57) | 2.83 (0.55) | 2.90 (0.61) | 2.89 (0.51) | 2.94 (0.61) | 3.01 (0.67) |  |  |  |  |
| SVRI (dynes sec/^-1^ cm^-5^ m^2^) | **Paracetamol** | 1919 (448) | 1842 (425) | 1740 (435) | 1840 (401) | 1891 (364) | 1908 (403) | 1891 (422) | 1926 (519) | 1951 (358) | 0.64 | -114 to 162 | 0.72 | 0.72 |
|  | **Saline** | 1847 (636) | 1862 (739) | 1788 (646) | 1749 (492) | 1845 (529) | 1804 (449) | 1794 (398) | 1749 (511) | 1754 (507) |  |  |  |  |
